# Using ‘Omic Approaches to Compare Temporal Bacterial Colonization of *Lolium perenne*, *Lotus corniculatus*, and *Trifolium pratense* in the Rumen

**DOI:** 10.3389/fmicb.2018.02184

**Published:** 2018-09-19

**Authors:** Christopher L. Elliott, Joan E. Edwards, Toby J. Wilkinson, Gordon G. Allison, Kayleigh McCaffrey, Mark B. Scott, Pauline Rees-Stevens, Alison H. Kingston-Smith, Sharon A. Huws

**Affiliations:** ^1^Institute of Biological, Environmental and Rural Sciences, Aberystwyth University, Aberystwyth, United Kingdom; ^2^Laboratory of Microbiology, Wageningen University & Research, Wageningen, Netherlands; ^3^The Roslin Institute, University of Edinburgh, Midlothian, United Kingdom; ^4^School of Biological Sciences, Medical Biology Centre, Queen’s University Belfast, Belfast, United Kingdom

**Keywords:** microbiome, perennial ryegrass, birds foot trefoil, red clover, 16S rRNA gene, FTIR, CowPI, rumen

## Abstract

Understanding rumen plant–microbe interactions is central for development of novel methodologies allowing improvements in ruminant nutrient use efficiency. This study investigated rumen bacterial colonization of fresh plant material and changes in plant chemistry over a period of 24 h period using three different fresh forages: *Lolium perenne* (perennial ryegrass; PRG), *Lotus corniculatus* (bird’s foot trefoil; BFT) and *Trifolium pratense* (red clover; RC). We show using 16S rRNA gene ion torrent sequencing that plant epiphytic populations present pre-incubation (0 h) were substantially different to those attached post incubations in the presence of rumen fluid on all forages. Thereafter primary and secondary colonization events were evident as defined by changes in relative abundances of attached bacteria and changes in plant chemistry, as assessed using Fourier transform infrared (FTIR) spectroscopy. For PRG colonization, primary colonization occurred for up to 4 h and secondary colonization from 4 h onward. The changes from primary to secondary colonization occurred significantly later with BFT and RC, with primary colonization being up to 6 h and secondary colonization post 6 h of incubation. Across all 3 forages the main colonizing bacteria present at all time points post-incubation were *Prevotella*, *Pseudobutyrivibrio*, *Ruminococcus*, *Olsenella*, *Butyrivibrio*, and *Anaeroplasma* (14.2, 5.4, 1.9, 2.7, 1.8, and 2.0% on average respectively), with *Pseudobutyrivibrio* and *Anaeroplasma* having a higher relative abundance during secondary colonization. Using CowPI, we predict differences between bacterial metabolic function during primary and secondary colonization. Specifically, our results infer an increase in carbohydrate metabolism in the bacteria attached during secondary colonization, irrespective of forage type. The CowPI data coupled with the FTIR plant chemistry data suggest that attached bacterial function is similar irrespective of forage type, with the main changes occurring between primary and secondary colonization. These data suggest that the sward composition of pasture may have major implications for the temporal availability of nutrients for animal.

## Introduction

It is predicted that the human population is going to double by 2050, rising to 9 billion (Foresight, 2011). Consequently, consumption of milk and red meat will likely double when compared with levels recorded at the beginning of the 21st century (Food and Agriculture Organization [FAO], 2006). Ruminants typically utilize only approximately 30% of their nitrogen intake to produce meat and milk, the rest being lost as urea or ammonia (Kingston-Smith et al., 2010). The urea and ammonia can be broken down by soil microbes into nitrous oxide, which is a potent greenhouse gas that contributes to climate change. Alternatively, they can leach into the environment where they cause eutrophication and thus have disastrous effects on aquatic ecosystems (Kingston-Smith et al., 2010). Thus, there is considerable incentive to reduce nitrogen losses from grazing ruminant livestock, and understanding ruminant digestion of plant material is paramount to increasing the efficiency of utilization by the animal.

The colonization of perennial ryegrass (PRG) is rapid (less than 5 min) and biofilms prevale from early on in colonization (Cheng et al., 1980; Miron et al., 2001; Russell and Rychlik, 2001; Koike et al., 2003; Edwards et al., 2007, 2008a,b; Huws et al., 2013, 2014, 2016; Mayorga et al., 2016). We also demonstrated that colonization of fresh PRG is a biphasic process, with primary (up to 4 h) and secondary (after 4 h) phases being evident as based on changes in bacterial diversity and plant chemical composition (Mayorga et al., 2016). We have also reported that the attached bacteria were low in endoglucanse capability, a factor that may impede the breakdown of plant cellulose/hemicellulose, resulting potentially in low bioavailability of nutrients for the ruminant (Mayorga et al., 2016). Nonetheless, it is unlikely that ruminants ingest perennial ryegrass alone as pasture, and more typically animals graze on mixed swards. Therefore, understanding bacterial colonization of other sward species is required to form a holistic understanding of the rumen plant–microbe interactome.

The aim of this study was to further our understanding of rumen plant-microbe interactions by comparing rumen bacterial colonization of three common forage species. Bacterial colonization of *Lolium perenne* (Perennial ryegrass; PRG), *Lotus corniculatus* (Birds Foot Trefoil; BFT) and *Trifolium pratense* (Red Clover; RC) was assessed over a period of 24 h using 16S rRNA gene sequencing, prediction of bacterial function was made using CowPI, and changes in plant chemistry was monitored by FTIR spectroscopy. All plant matter used in this study was fresh forage as 60% of the United Kingdom agricultural land is used for the growth of fresh forage for grazing purposes, with similar trends across Europe. This study advances current understanding of plant–bacterial interactions in the rumen, and in turn helps identification of key bottlenecks, guiding technological developments for the sustainable increase of ruminant production.

## Materials and Methods

### Growth of Plant Matter and Chemical Analysis

For this study greenhouse grown material was used to minimize variation in growth conditions of the plants, thus limiting any environmental variables which may affect colonization in order to obtain preliminary data comparing colonization of each plant type. PRG (*Lolium perenne* cv. AberDart) was grown from seed in plastic trays (length 38 cm × width 24 cm × depth 5 cm) containing compost (John Innes no. 3). BFT (*Lotus corniculatus* cv. Maitland) and RC (*Trifolium pratense* cv. Milvus) were grown from scarified seeds in 77 plug trays containing compost (John innes no.3). BFT seedlings were thinned to 1 plant per plug after 2 weeks, transferred to 3.5″ pots after 5 weeks and transferred to 5″ pots after 9 weeks. RC seedlings were thinned to 1 plant per plug after 2 weeks, transferred to 3.5″ pots after 6 weeks and transferred to 5″ pots after 12 weeks. BFT and RC were trimmed back at 3, 10, 11, and 13 weeks with RC additionally being trimmed back at week 5. All plants were housed in a greenhouse under natural irradiance with additional illumination provided to maintain a minimum of 10 h photoperiod. The temperature was kept at 20/10°C day/night cycle and the plants were watered daily, with low level irrigation (1 × 30 s daily) applied to BFT and RC between week 11 and harvesting at week 16. Growth of plant material was co-ordinated so that each forage type could be harvested together on the same experimental day.

Before each experiment sub-samples (*n* = 3) of each plant type were taken and fozen at -20°C then freeze-dried for subsequent chemical composition analysis. Water-soluble carbohydrate (WSC) concentration of forages was determined spectrophotometrically using anthrone in sulphuric acid on a Technicon Autoanalyser (Technicon Corporation) (Thomas, 1977). Neutral-detergent fiber was determined as described by Van Soest et al. (1991) and the Tecator Fibretec System (Tecator Limited)). Acid-detergent fiber was analyzed according to the method of Van Soest and Wine (1967) using the Tecator Fibretec System (Tecator Limited). Nitrogen (N) concentration of diets was analyzed by combustion at 550°C using a LECO FP-428 analyzer (LECO Corporation). Moisture content of samples was determined by gravimetric loss after oven drying.

### Rumen Fluid Inoculum

Rumen fluid was obtained from three rumen-cannulated cows under the authority of licenses under the UK Animal Scientific Procedures act 1986. All animal associated research must have approval by the Aberystwyth University ethics committee (Aberystwyth University policy on animal experiments can be found here https://www.aber.ac.uk/en/media/departmental/rbi/staff-students/ethics/Experimental-work-involving-animals-at-Aberystwyth-University-En-v-2.pdf). Cows were grazed mainly on fresh forage to allow adaptation beforehand, but with some PRG silage input before the experiment. On the day of each experiment, rumen content was collected 2 h post-feeding and strained through two layers of muslin. The rumen fluid contained both planktonic and attached microbes as small forage fragments pass through the muslin. Use of strained rumen fluid as inoculum is commonplace when studying the rumen microbiome *in vitro* (Ramos-Morales et al., 2017) and we have previously demonstrated that this *in vitro* technique results in similar results to those obtained *in vivo* (Mayorga et al., 2016). Equal volumes of the strained rumen fluid from the three cows were pooled, held under CO_2_ and kept at 39°C until use in the *in vitro* incubations.

### *In vitro* Incubations

After 6 weeks of growth, PRG was cut at 3 cm above the soil, washed in distilled water and cut into 1 cm sections. BFT and RC plants were harvested after 16 weeks of growth, where upon the leaves were washed in distilled water and cut in half. Each forage type (7.5 g fresh weight) was placed in Duran bottles (250 mL) together with anaerobic incubation buffer (135 mL at 39°C; Van Soest, 1967) and rumen fluid inoculum (15 mL). This method is simple but has been shown to replicate colonization events seen in the rumen previously, making it a good model system to study colonization (Mayorga et al., 2016).

The bottles were incubated at 39°C in a horizontally rotating rack set at 100 rpm (Incubator- shaker, LA engineering, United Kingdom). Three bottles for each substrate were harvested at 1, 2, 3, 4, 6, 8, 12, and 24 h. The 0 h samples were taken pre-addition of rumen fluid as the rumen bacteria rapidly attach and capturing ‘true’ 0 h post-rumen fluid addition would not be possible. The contents of each bottle was destructively harvested, with the plant material collected by vacuum filtration through filter paper (11 μm^2^ pore size; ^®^QL100, Fisher Scientific, Leicestershire, United Kingdom). Retained plant material was washed with 50 mL phosphate buffered saline (PBS) to remove any loosely attached bacteria. The attached bacteria were removed from the plant material by overnight incubation at 4°C in glutaraldehyde (3% v/v in PBS). The absence of attached bacteria on the plant material was confirmed by transmission electron microscopy as described by Mayorga et al. (2016) (data not shown). The bacterial suspension underwent centrifugation (10,000 ×*g*, 10 min) and the pellet produced was freeze dried for 16S rRNA gene sequencing. Following removal of the colonizing bacteria, the plant residue was recovered by squeezing the contents through a layer of muslin to remove the majority of the surface liquid followed by freeze-drying. This freeze-dried plant matter was weighed to allow the calculation of percentage dry matter degradation. The plant material was finely ground in liquid nitrogen with a reciprocal shaking system (particle size < 0.05 mm) for FTIR analysis. Experiments were repeated on 3 consecutive days with *n* = 3 during each experiment resulting in a total of *n* = 9 for each forage and incubation time.

### Extraction of DNA

Bacterial DNA from the freeze-dried cell pellets were extracted using the BIO101 FastDNA^®^ SPIN Kit for soil (Qbiogene, Cambridge, United Kingdom) alongside a FastPrep^®^ cell disrupter instrument (Bio101, ThermoSavant, Qbiogene) following the manufacturer’s instructions with the exception that the samples were processed for 3 × 30 s at speed 6.0 in the FastPrep instrument. The quality and quantity of the DNA was assessed using an Epoch microplate spectrophotometer (Biotek, Bedfordshire, United Kingdom).

### 16S rRNA Gene Sequencing

16S rRNA gene ion torrent sequencing was completed for the 3 plant substrates for each time point (0, 1, 2, 3, 4, 6, 8 12, and 24 h). Ion torrent based 16S rRNA gene sequencing is used extensively to monitor the rumen microbiome (see de la Fuente et al., 2014; Belanche et al., 2017; Indugu et al., 2017 as examples). Amplicons of the V6-V8 variable regions of the bacterial 16S rRNA gene were generated for each sample in triplicate using the primers AACAGGATTAGATACCCTG – 799F2 and CGTCRTCCCCRCCTTCC – 1177R (Belanche et al., 2017) containing unique ion torrent adaptors. These primers were chosen as they have been previously validated in order to reduce PCR amplification of chloroplastic 16S rRNA gene, which would result in most sequences being those from chloroplast 16S rRNA gene and less actual bacterial sequences being available for analysis (Edwards et al., 2007; Huws et al., 2007). PCR was performed using PCRBIO hifi taq mix (PCRBiosystems, London, United Kingdom) using approximately 100 ng DNA template. PCR cycling was conducted as follows: denaturing at 95°C for 3 min followed by 25 cycles of 95°C for 15 s, 60 for 16 s, 72°C for 15 s and a final chain elongation of 72°C for 5 min. PCR products were initially verified by agarose gel electrophoresis on a 1.0% (w/v) agarose gel for 1 h, 120 V, and 80 MA in 1% TAE (Tris base, acetic acid and EDTA) buffer. Subsequently, triplicate replicates of the PCR for each sample were pooled, and 30 μl of each were subjected to electrophoresis on a 2.0% (w/v) agarose gel for 2 h, 120 V, and 80 MA in 1% TAE buffer. The bands were observed and excised using a dark reader transilluminator (Clare Chemical Research, Dolores, CO, United States), and amplicons from the cut bands obtained using the Isolate II PCR and Gel Kit (Bioline, London, United Kingdom). Using the Agilent High Sensitivity Assay Kit (Agilent Technologies, Santa Clara., CA, United States) the purified amplicons were verified and quantified, and were then sequenced using the Ion Torrent PGM sequencer utilizing the Ion PGM Template OT2 400 and Ion PGM Hi-Q Sequencing kits (Life Technologies Ltd., Paisley, United Kingdom). The sequences are deposited in the European Nucleotide Archive under the project number PRJEB25753.

### Sequence Analysis

16S rRNA gene sequences were processed as described by Wilkinson et al. (2017). Essentially, the CD-HIT-OTU pipeline (Li et al., 2012) was used to denoise the sequences and remove low quality sequences, pyrosequencing errors and chimeras. The sequences were then clustered into Operational Taxonomic Units (OTUs) at 97% identity using the CD-HIT-OTU pipeline. OTUs with less than 10 reads were not included as they were likely to be sequencing artifacts. MOTHUR (Schloss et al., 2009) was used to classify the OTUs against the Greengenes 16S rRNA gene database (version 13.5). Calculations of alpha diversity and beta dispersion were performed using the phyloseq Bioconductor package in R, multivariate ANOVA of bray- curtis distance matrices were assessed by 1,000 permutations and corrected using the Bonferroni method (McMurdie and Holmes, 2013). The genomic and metabolic potential represented by the attached bacteria at each timepoint on each forage type was predicted using CowPI (Wilkinson et al., 2018), which is a rumen microbiome focussed version of Phylogenetic Investigation of Communities by Reconstruction of Unobserved States 165 (PICRUSt) (Langille et al., 2013).

### FTIR Spectroscopy

Before analysis, the 3 biological replicates for each timepoint and forage, within each experiment, were pooled. Mid infrared spectra were obtained for each forage and time point by attenuated total reflectance (ATR) using a Bruker Equinox 55 spectrometer (Bruker Optics Ltd., Coventry, United Kingdom) equipped with a deuterated tryglycine sulfate detector and a Golden Gate ATR accessory (Specac., Ltd., Orpington, United Kingdom). Spectra over the range 4000–500 cm^-1^ were acquired as a mean of 32 scans and at a spectral resolution of 4 cm^-1^ using OPUS software (version 4.2, Bruker Optics Ltd., Coventry, United Kingdom).

### Statistical Analysis

Principal component analysis ordination plots of OTU data were constructed using the Phyloseq program for R (McMurdie and Holmes, 2013). Constrained analysis of proximities (CAP) ordination plots of OTU data were also constructed and multiple analysis of variance (MANOVA) performed on this data using adonis2 in R vegan (Oksanen et al., 2008). On a genus level, significant differences between forage types (F) and timepoints (T) as well as F^∗^T interactions were identified using two-way analysis of variance (ANOVA) using GenStat (Payne et al., 2007). Where significant differences in attached bacterial genera were detected on an F and/or T basis, histograms were drawn using Microsoft excel to visualize the differences further. Histograms were not drawn for genera that had a proportional percentage read average < 0.1% across the timepoints or only showed a significant decrease in proportion between 0 and 1 h. Genus data showing a significant interaction between F and T were also not plotted. Statistical Analysis of Metagenomic Profiles (STAMP) was used to analyze the CowPI data and to calculate principal components based on Euclidean distance between samples for the taxonomic PCoA plots. The STAMP analyzed samples were blocked by forage type, experiment number and timepoint, and subjected to an ANOVA (multiple groups) with 1,000 permutations, Tukey–Kramer *post hoc* analysis results were corrected for multiple testing using the Bonferroni method. For the PCoAs, 0 h data were omitted as they skewed the plots and reduced the ability to see the changes in diversity over incubation time. Using OPUS software, the FTIR spectra were converted into text files and imported to Matlab (version 6.5.1) for statistical and chemometric analysis using the Matlab Statistics Toolbox (Version 4, The Mathworks, Cambridge, United Kingdom) and the PLS toolbox (version 8.2.1, Eigenvector Research Inc., Manson, WA, United States). Spectra were analyzed for underlying structure correlating with incubation time and forage type using principal component analysis (PCA) (Sheng et al., 2006). The spectra recorded for the 24 h samples of PRG and RC were omitted from the analysis as they dominated the model. The PCA scores of relevant components (3 for PRG, 4 for clover and 5 for lotus) were also further analyzed by multivariate analysis of variance (MANOVA) to test for significant chemical changes in each forage over time using Genstat (Payne et al., 2007).

## Results

### Plant Chemical Composition and *in vitro* Degradation

Dry matter was similar for all plant types used, but total N was higher for BFT and RC compared with PRG (**Table 1**). Levels of WSC, NDF and ADF were higher for PRG compared with BFT and RC (**Table 1**). Recovered plant matter showed a decline in dry matter in all 3 of the forage types over time (**Table 2**). RC had the most rapid decline in plant dry matter shown most evidently at 3 h where RC had degraded by around 6 times more than PRG and BFT. The increased dry matter loss in RC was maintained up until 12 h, but by 24 h PRG showed the highest loss in dry matter. BFT had the slowest rate of degradation and by 24 h only 49.72% had degraded compared with 77.72 and 89.97% for RC and PRG respectively (*P* = 0.003)

**Table 1:** Mean chemical composition [g/Kg dry matter (DM)] of the experimental diets.

	*L. perenne*	*L. corniculatus*	*T. pratense*
DM	924.2	922.8	911.0
Total Nitrogen (N)	22.5	46.1	42.2
Water-soluble carbohydrate (WSC)	176.7	28.5	32.4
Neutral-detergent fiber (NDF)	413.4	255.2	177.2
Acid detergent fiber (ADF)	239.3	122.6	105.5

**Table 2:** Plant dry matter degradation (g dry matter lost from 100 g of initial dry matter) following *in vitro* ruminal incubation of *Lolium perenne, Lotus corniculatus*, and *Trifolium pratense* over time.

Time (h)	*L. perenne*	*L. corniculatus*	*T. pratense*	SED	*P*
1	2.24	1.52	1.64	0.40	0.297
2	4.88	1.53	7.95	3.39	0.238
3	5.01	3.17	23.00	6.66	0.073
4	15.86	4.98	22.30	11.09	0.380
6	18.00	12.73	29.10	5.34	0.084
8	30.35	15.79	32.80	10.42	0.251
12	34.65	26.78	39.62	13.28	0.641
24	89.97	49.72	77.72	5.09	0.003

### Bacterial Colonization

**FIGURE 1:**
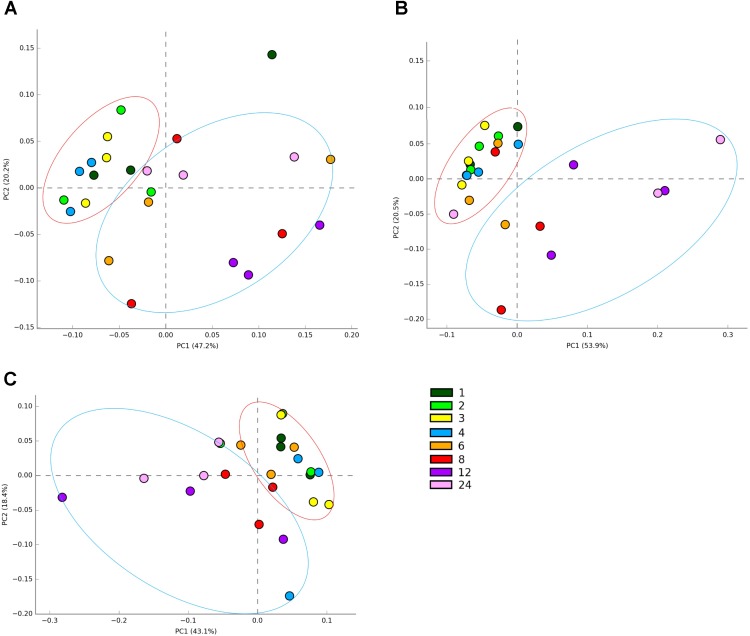
Principal coordinates analysis (PCoA) of the attached bacterial genera present on *Lolium perenne* **(A)**, *Lotus corniculatus* **(B)**, and *Trifolium pratense* **(C)** over the 1–24 h incubation period. Numbers correspond with incubation time in hours, with *n* = 3 for each time point. Red circles show early colonization events and blue circles secondary colonization events. Outliers can be seen outside the circles denoted.

In terms of sequencing depth pre-quality control, we obtained on average 88,019 reads/sample and 57,964 remained post filtering, with an average length of 304 bp pertaining to an average of 1548 OTUs (**Supplementary Table S1**). PCoA plots of OTU abundances showed that there were changes in colonization on all the plant types over the 24 h incubation period. When PCoA plots for all samples were plotted together, it was difficult to see any patterns in colonization events (**Supplementary Figure S1**), therefore, the data is shown per forage type in the main manuscript (**Figures 1A–C**). The PRG attached bacteria clustered at 1, 2, 3, 4 h and then a shift was observed around 6, 8, 12, and 24 h post colonization (**Figure 1A**). The bacteria associated with BFT and RC showed clustering at 1, 2, 3, 4, 6 h and then a shift was observed at 8, 12, and 24 h post colonization (**Figures 1B,C** respectively). The CAP plots showed very similar results to the PCoA plots with slightly more separation based on the time points evident (**Supplementary Figure S2**). MANOVA results showed that plant type affected colonization (*P* < 0.001) and with each plant type, time had a significant affect on the attached bacterial diversity (*P* < 0.001 for RC and *P* < 0.05 for PRG and BFT).

The sequences showed that on a phylum level, the main difference occurred from pre-incubation (0 h) to post incubation (1 h) (data not shown). Before incubation (0 h) Proteobacteria, Actinobacteria, Firmicutes, and Bacteroidetes dominate, in descending order, in the presence of all forages tested. Then post-incubation (1 h) the abundance of Firmicutes, Tenericutes and Bacteroidetes were on average higher (data not shown). On a family level, as with the phylum, the main differences were between pre (0 h) and post (1 h onward) incubation in the presence of rumen fluid. Pre-incubation (0 h) the dominant attached bacterial families were *Burkholderiaceae*, *Comamonadaceae*, *Enterobacteriaceae, Lachnospiraceae, Microbacteriaceae, Prevotellaceae*, and *Xanthamonadaceae* on all forages. From 1 h onward the dominant families were *Coriobacteriaceae Lachnospiraceae*, *Prevotellaceae*, *Ruminococcaceae*, and *Veillonellaceae*, with decreases in *Burkholderiaceae*, *Comamonadaceae, Enterobacteriaceae, Microbacteriaceae* and *Xanthamonadaceae* observed (data not shown)

**FIGURE 2:**
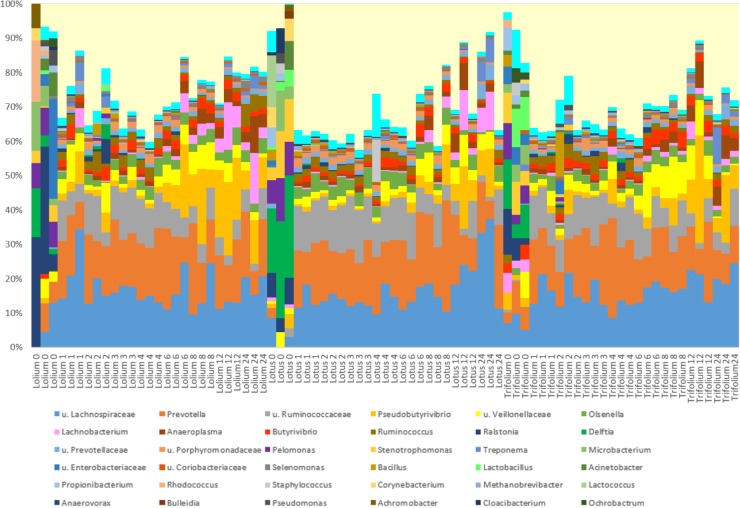
Relative abundances of the colonizing bacterial genera attached to *Lolium perenne, Lotus corniculatus*, and *Trifolium pratense* at different timepoints of *in vitro* rumen incubation. The x axis describes the type of forage and incubation time. The prefix u. signifies that the genus could not be classified with 85% confidence so family level of classification is shown. Minor genera representing < 0.1% of total reads on average across time points and forage type were removed.

**FIGURE 3:**
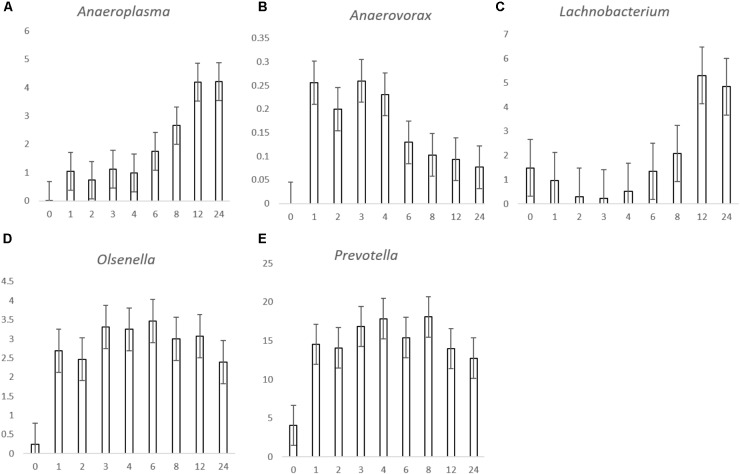
Histograms showing the proportion of bacterial genera *Anaeroplasma* **(A)**, *Anaerovorax* **(B)**, *Lachnobacterium* **(C)**, *Olsenella* **(D),** and *Prevotella* **(E)** attached to *Lolium perenne, Lotus corniculatus*, and *Trifolium pratense* post rumen-like *in vitro* incubation over time. The *X* axis corresponds with the number of hours post-incubation and the *y* axis shows the percentage of relative read abundance. Values are given as the mean of the 3 forages (*n* = 9) for each timepoint and error bars are given as standard error of mean.

**FIGURE 4:**
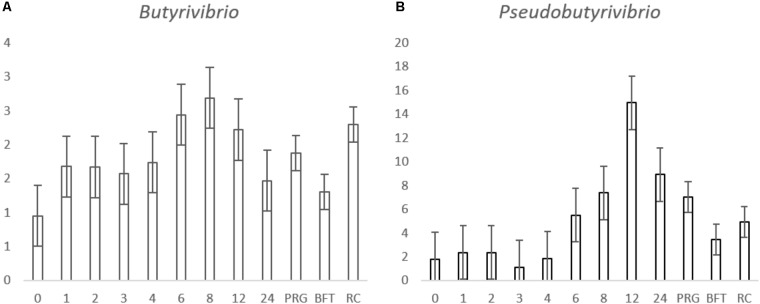
Histograms showing the proportion of *Butyrivibrio* and **(A)**, *Pseudobutyvibrio* **(B)** attached to *Lolium perenne* (PRG), *Lotus corniculatus* (BFT) and *Trifolium pratense* (RC) post rumen-like *in vitro* incubation over time. The numbers in the x axis correspond with the number of hours post-incubation and the letters correspond with the forage type comparisons. The y axis shows the percentage relative abundance. Values for the time comparison are given as the mean of the 3 forages (*n* = 9) at each timepoint, values for the forage type comparison are given as a mean of all time points in the forage group (*n* = 27) and error bars are given as standard error of mean.

For the genus level data, we removed data for genera that pertained to < 0.1% of total reads on an average basis across time points and forage type to ease interpretation (**Figure 2** and **Table 3**). We did, however, plot the data for the minor genera (those < 0.1%) but patterns of colonization were not apparent within these minor colonizers (**Supplementary Figure S3**), therefore we discuss the major attached bacterial fraction (>0.1%) from this point forward. On the genus level, the most abundant bacteria pre-incubation (0 h) were *Delftia, Lactobacillus, Lactococcus Microbacterium, Mitsuokella, Prevotella, Pelomonas, Ralstonia, Stenotrophomonas* (**Figure 2** and **Supplementary Table S2**). Post incubation (from 1 h onward) the dominant attached genera were *Anaeroplasma, Butyrivibrio, Olsenella, Prevotella, Pseudobutyrivibrio*, and *Ruminococcus* (**Figure 2**, **Table 3**, and **Supplementary Table S2**). The abundance of PRG-attached *Acineobacter*, *Microbacterium* and *Pelomonas* decreased post incubation (*P* < 0.05) (0–1 h). On BFT and RC there was a significant reduction between 0 and 1 h in the abundance of *Delftia*, *Lactobacillius*, *Pelomnas*, *Propionibacterium*, and *Stenotrophomonas* (*P* < 0.05). Reductions in BFT-attached *Microbacterium* and *Ochrobactrum* (*P* < 0.05) was also seen between 0 and 1 h of incubation. Between 0 and 1 h, increases in attached *Anaeroplasma* abundance was seen on all forages (*P* < 0.05) with further increases after 6 h (**Figure 3A** and **Supplementary Table S2**) suggesting that it is a primary colonizer which has a further role in secondary colonization. *Anaerovorax* proportional read abundances were higher during primary colonization and decreased during secondary colonization on all forages (**Figure 3B** and **Supplementary Table S2**). *Butyrivibrio, Olsenella*, and *Prevotella* all showed a pattern of significant increase between 0 and 1 h but no further significant changes post incubation (**Figures 3D,E**, **4A** and **Supplementary Table S2**). *Lachnobacterium* and *Pseudobutyvibrio* showed a significant increase at around 12 h (**Figures 3C**, **4B** and **Supplementary Table S2**) suggesting that these genera have an important role in the final stages of the breakdown of plant matter. Between the forage types the only significant differences were between *Butyvibrio* and *Pseudobutyvibrio*, which in both cases had a reduced abundance when attached to BFT compared with PRG and RC (**Figures 4A,B**, **Table 3**, and **Supplementary Table S2**). Alpha diversity data showed no difference in bacterial diversity when all forages were compared, irrespective of the method used (**Supplementary Figure S4**).

**Table 3:** Effects of forage type (F) and incubation time (h) on bacterial colonization of *Lolium perenne, Lotus corniculatus* and *Trifolium pratense* after *in vitro* rumen incubation.

Genus	*P*-value			SED		
	F	T	F^∗^T	F	T	F^∗^T
*Acholeplasma*	NS	0.01	NS	0.02	0.04	0.07
*Achromobacter*	NS	NS	NS	0.22	0.38	0.65
*Acidovorax*	NS	NS	NS	0.07	0.12	0.20
*Acinetobacter*	NS	<0.001	NS	0.31	0.53	0.92
*Agrococcus*	NS	NS	NS	0.06	0.10	0.17
*Alcaligenes*	NS	NS	NS	0.01	0.02	0.04
*Alloprevotella*	NS	0.013	NS	0.01	0.01	0.02
*Amnibacterium*	NS	NS	NS	0.00	0.01	0.01
*Anaerobacterium*	NS	NS	NS	0.01	0.01	0.02
*Anaerofustis*	NS	0.012	0.045	0.00	0.01	0.01
*Anaeroplasma*	NS	<0.001	NS	0.39	0.67	1.16
*Anaerorhabdus*	NS	NS	NS	0.01	0.01	0.02
*Anaerovibrio*	NS	0.013	0.008	0.02	0.04	0.07
*Anaerovorax*	NS	<0.001	NS	0.03	0.05	0.08
*Aneurinibacillus*	NS	NS	NS	0.01	0.01	0.02
*Arthrobacter*	0.046	NS	NS	0.01	0.01	0.02
*Atopobium*	NS	0.008	NS	0.01	0.02	0.03
*Bacillus*	NS	NS	NS	0.43	0.74	1.27
*Blautia*	NS	NS	NS	0.06	0.10	0.17
*Brachybacterium*	NS	NS	NS	0.01	0.02	0.04
*Brevibacillus*	NS	NS	NS	0.02	0.03	0.05
*Brevundimonas*	NS	NS	NS	0.05	0.08	0.14
*Bulleidia*	NS	NS	NS	0.07	0.12	0.21
*Burkholderia*	0.041	0.041	0.029	0.01	0.01	0.02
*Butyrivibrio*	0.001	0.011	NS	0.26	0.45	0.78
*Chryseobacterium*	NS	0.005	<0.001	0.03	0.06	0.10
*Cloacibacterium*	NS	NS	NS	0.21	0.36	0.63
*Coprococcus*	NS	<0.001	0.021	0.02	0.04	0.06
*Corynebacterium*	NS	<0.001	0.034	0.22	0.38	0.66
*Curtobacterium*	NS	NS	NS	0.00	0.00	0.01
*Delftia*	0.002	<0.001	<0.001	0.56	0.97	1.68
*Denitrobacterium*	NS	0.032	NS	0.01	0.02	0.04
*Diaphorobacter*	NS	NS	NS	0.05	0.08	0.14
*Eubacterium*	NS	0.04	NS	0.00	0.01	0.01
*Exiguobacterium*	NS	NS	NS	0.01	0.02	0.04
*Ezakiella*	NS	NS	NS	0.04	0.06	0.11
*Facklamia*	NS	NS	NS	0.00	0.01	0.01
*Fibrobacter*	NS	NS	NS	0.01	0.03	0.04
*Kandleria*	NS	0.03	NS	0.00	0.01	0.01
*Kocuria*	NS	NS	NS	0.19	0.33	0.57
*Lachnobacterium*	NS	<0.001	NS	0.67	1.17	2.02
*Lactobacillus*	NS	<0.001	0.007	0.34	0.59	1.01
*Lactococcus*	NS	NS	NS	0.46	0.79	1.37
*Leptotrichia*	NS	NS	NS	0.09	0.16	0.27
*Leuconostoc*	NS	NS	NS	0.00	0.00	0.01
*Limnobacter*	NS	NS	NS	0.01	0.01	0.02
*Lysinibacillus*	NS	NS	NS	0.03	0.05	0.09
*Massilia*	0.018	NS	0.024	0.10	0.18	0.31
*Methylobacterium*	NS	NS	NS	0.00	0.00	0.01
*Microbacterium*	NS	<0.001	NS	0.47	0.81	1.40
*Mitsuokella*	NS	NS	NS	0.08	0.14	0.25
*Mogibacterium*	NS	NS	NS	0.01	0.01	0.02
*Moryella*	NS	<0.001	NS	0.01	0.02	0.03
*Mycobacterium*	NS	0.043	NS	0.01	0.02	0.04
*Nocardiopsis*	NS	NS	NS	0.00	0.00	0.01
*Noviherbaspirillum*	NS	NS	NS	0.01	0.01	0.02
*Ochrobactrum*	NS	0.001	NS	0.13	0.23	0.40
*Oligosphaera*	NS	NS	NS	0.00	0.01	0.01
*Olsenella*	NS	<0.001	NS	0.33	0.56	0.98
*Oscillibacter*	NS	NS	NS	0.01	0.01	0.02
*Paenibacillus*	NS	NS	NS	0.05	0.09	0.15
*Paraprevotella*	NS	NS	NS	0.00	0.01	0.01
*Pelomonas*	NS	<0.001	NS	0.26	0.45	0.78
*Prevotella*	NS	<0.001	NS	1.50	2.60	4.51
*Propionibacterium*	NS	<0.001	NS	0.20	0.35	0.61
*Pseudobutyrivibrio*	0.028	<0.001	NS	1.30	2.25	3.90
*Pseudoflavonifractor*	NS	NS	NS	0.00	0.01	0.01
*Pseudomonas*	NS	0.009	NS	0.16	0.27	0.47
*Ralstonia*	0.048	<0.001	0.003	0.99	1.71	2.97
*Rhodococcus*	NS	NS	NS	0.52	0.91	1.57
*Roseburia*	NS	NS	NS	0.00	0.01	0.01
*Rothia*	NS	NS	NS	0.07	0.12	0.20
*Ruminococcus*	0.017	<0.001	0.047	0.25	0.44	0.76
*Saccharofermentans*	NS	0.007	NS	0.01	0.02	0.03
*Selenomonas*	<0.001	<0.001	0.002	0.08	0.14	0.24
*Slackia*	NS	<0.001	NS	0.01	0.01	0.02
*Sphaerochaeta*	NS	<0.001	NS	0.02	0.03	0.05
*Sphingomonas*	0.031	NS	0.026	0.06	0.10	0.17
*Staphylococcus*	NS	NS	NS	0.39	0.67	1.16
*Stenotrophomonas*	NS	<0.001	0.007	0.35	0.61	1.05
*Streptococcus*	NS	NS	NS	0.09	0.15	0.26
*Streptophyta*	0.005	NS	NS	0.07	0.12	0.20
*Succiniclasticum*	NS	0.007	NS	0.01	0.02	0.03
*Succinivibrio*	NS	NS	NS	0.02	0.04	0.07
*Thermoactinomyces*	NS	NS	NS	0.01	0.01	0.02
*Treponema*	NS	<0.001	NS	0.01	0.01	0.02
*Turicibacter*	NS	NS	NS	0.00	0.01	0.01
*Weissella*	NS	0.012	NS	0.02	0.04	0.07
*Williamsia*	NS	NS	NS	0.00	0.01	0.01
unclassified	0.02	<0.001	NS	1.83	3.17	5.48
u.Acidaminococcaceae	NS	NS	NS	0.00	0.00	0.01
u.Bacillaceae	NS	NS	NS	0.10	0.17	0.29
u.Comamonadaceae	0.033	0.002	<0.001	0.02	0.03	0.05
u.Coriobacteriaceae	NS	0.011	NS	0.07	0.12	0.21
u.Desulfovibrionaceae	NS	<0.001	0.002	0.01	0.02	0.04
u.Enterobacteriaceae	NS	0.009	NS	0.84	1.46	2.53
u.Erysipelotrichaceae	0.043	0.006	0.011	0.01	0.02	0.03
u.Eubacteriaceae	NS	NS	NS	0.01	0.01	0.02
u.Flavobacteriaceae	NS	NS	NS	0.01	0.01	0.02
u.Lachnospiraceae	NS	<0.001	NS	1.38	2.39	4.14
u.Neisseriaceae	NS	NS	NS	0.08	0.15	0.25
u.Peptococcaceae	NS	0.008	0.036	0.01	0.01	0.02
u.Planctomycetaceae	NS	0.026	NS	0.02	0.03	0.05
u.Planococcaceae	NS	0.013	NS	0.04	0.07	0.12
u.Porphyromonadaceae	0.039	<0.001	NS	0.15	0.26	0.45
u.Prevotellaceae	NS	<0.001	NS	0.18	0.30	0.53
u.Rhodospirillaceae	NS	NS	NS	0.01	0.01	0.02
u.Ruminococcaceae	NS	<0.001	NS	0.78	1.36	2.35
u.Sutterellaceae	NS	NS	NS	0.01	0.01	0.02
u.Veillonellaceae	0.049	<0.001	NS	0.78	1.36	2.35

*F^∗^T shows if there are interactions between F and T. P-values were obtained using a one-way ANOVA and SED is standard error of the difference. Note that assignment to genus was based of 97% similarity but the prefix u. signifies that the genus could not be classified with 85% confidence so family level of classification is shown. NS is shown when data is non significant (*P* > 0.05).*

### Bacterial Function

**FIGURE 5:**
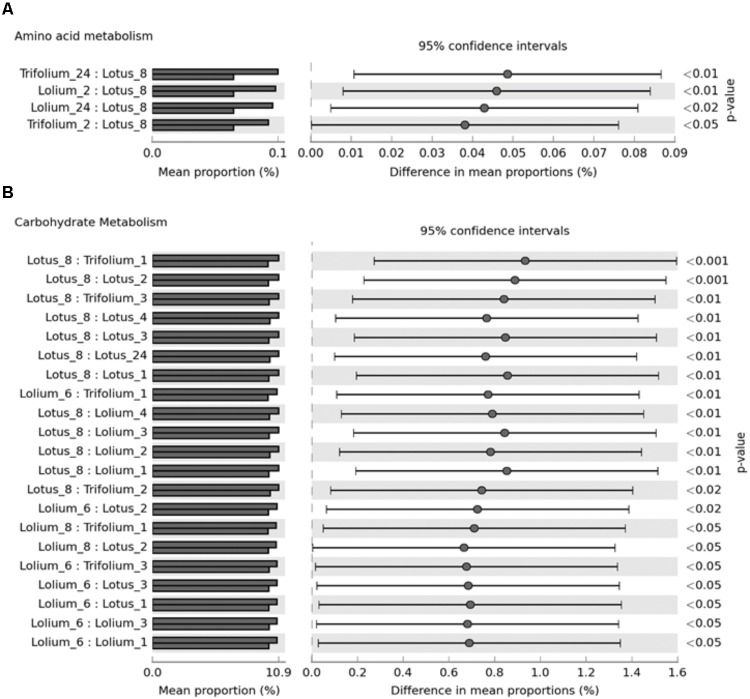
CowPI predicted metagenomes suggesting significant differences (*p* < 0.05) in amino acid metabolism **(A)** and carbohydrate metabolism **(B)** across *Lolium perenne, Lotus corniculatus*, and *Trifolium pratense* across different timepoints after an *in vitro* rumen incubation.

A total of 88% of the OTUs mapped to the Greengenes, thus the CowPI data is based on these. CowPI predictions are only shown for metabolic functions which were found to differ significantly in abundance when comparing forage type and incubation time (**Figure 5**). CowPI predictive analysis of the amino acid metabolism suggested that amino acid metabolism was higher within bacteria following 24 h of colonization on RC and PRG compared to those attached on BFT at 8 h post-incubation (**Figure 5A**). Amino acid metabolic capacity was also higher within PRG and RC attached bacteria at 2 h post colonization compared with those attached to BFT post 8 h of colonization (**Figure 5A**). Most functional differences were associated with the carbohydrate metabolism, with bacteria attached following 6 and 8 h of incubation showing higher carbohydrate metabolic capacity than those attached at earlier time points on all forages (**Figure 5B**).

### Plant Chemical Changes

**FIGURE 6:**
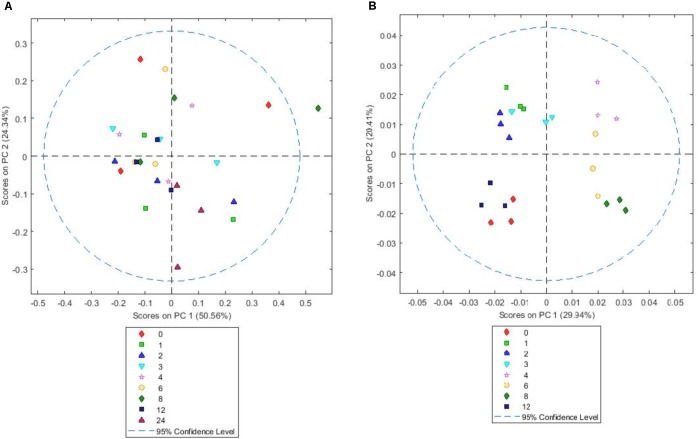
Simple score plot **(A)** and GLS weighted score plot **(B)** of principal components PC1 vs. PC2 for an FTIR spectrum of *Lolium perenne* plant matter after different times (hours) of *in vitro* rumen incubation. Please note that 24 h data are not shown in GLS weighted plot as they were clear outliers which skewed the plot.

**FIGURE 7:**
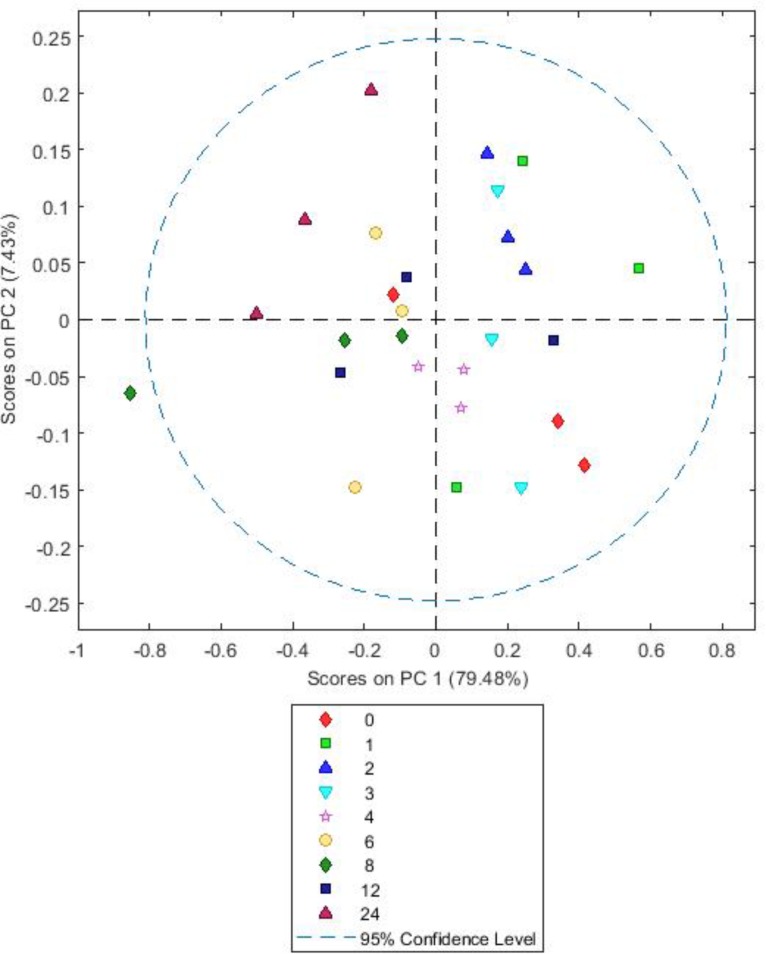
Simple score plot of principal components PC1 vs. PC2 for an FTIR spectrum of *Lotus corniculatus* plant matter after different times (hours) of *in vitro* rumen incubation.

**FIGURE 8:**
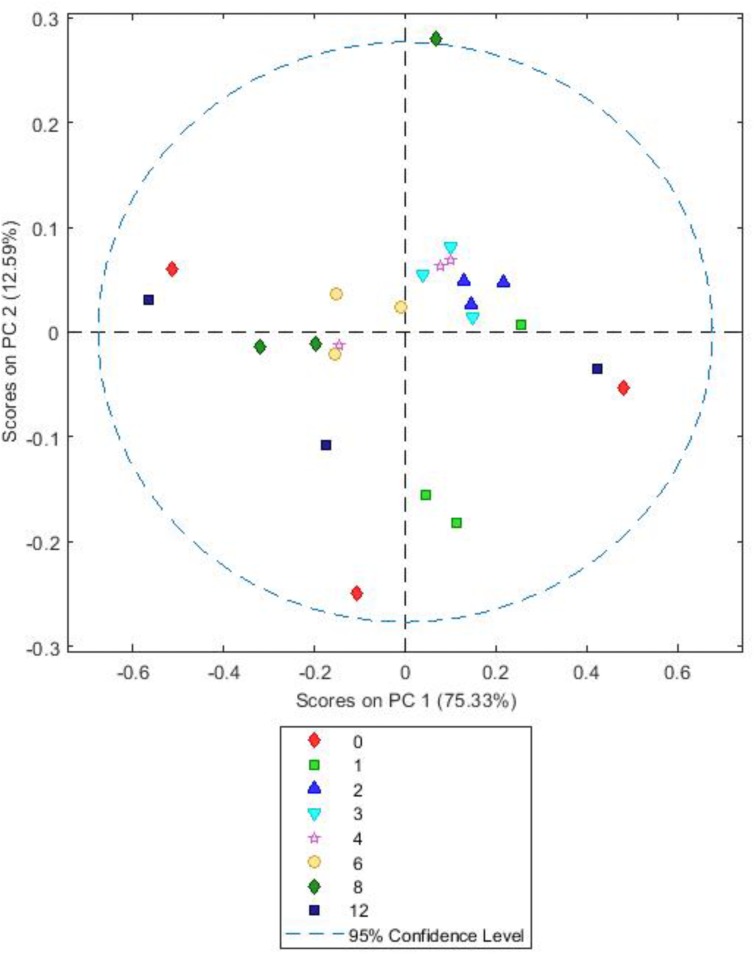
Simple score plot of principal components PC1 vs. PC2 for an FTIR spectrum of *Trifolium pratense* plant matter after different times (hours) of *in vitro* rumen incubation. Please note that 24 h data are not shown they were clear outliers which skewed the plot.

Analysis of the spectra comparing the different forage types showed there is clear clustering on PC1, accounting for 86% of the variation in the data, but no clustering or patterns on PC2, accounting for 4.19% of the data. Changes in PC1 are likely due to BFT and RC being dicots and PRG being a monocot. As a consequence, in order to see time associated changes each forage type was analyzed separately (**Figures 6–8**).

The simple PCA for PRG showed no real clustering or patterns, but inclusion of general least squares weighting (GLS) by class as a preprocessing step prior to PCA revealed distinct sample clusters (**Figures 6A,B**). General least squares weighting is a regression algorithm used to denoise data and clarify groupings in PCA. We used GLS weighting only on the PRG model as it did not lead to improved separation of groups with BFT or RC suggesting variance in the PRG data was different in nature to that in the BFT and RC data.

The scores on PC1 and 2 indicate that plant chemistry in the samples alters between 2 and 4 h, with 3 h being an intermediate as observed by Mayorga et al. (2016) (**Figure 6B**). This is most likely due to a change from primary to secondary colonization. The loadings (**Supplementary Figure S5**) show that changes correlated with polysaccharides (1200–900 cm^-1^) (Alonso-Simon et al., 2004); proteins (1310–1390 cm^-1^) (Schmitt and Flemming, 1998), fatty acids (2956–2850 cm^-1^), nucleic acids (1250 cm^-1^) and amides (1652–1648 cm^-1^). The sample scores on PC2 loadings become negative between 4 to 8 h, attributable to changes in polysaccharides (1000–950 cm^-1^) and fatty acids (2956–2850 cm^-1^). MANOVA Wilks’s lambda results also showed that the changes in plant chemistry seen as incubation time progressed were significant for PRG (*P* < 0.001).

Both BFT and RC PCA models showed highly similar loadings (**Supplementary Figures S6**, **S7**) and score plots (**Figures 7**, **8**). For both, A simple PCA models of the forages showed changes in plant chemistry on PC1 accounting for 79.48% (BFT) and 75.33% (RC) of the data. Timepoints 1–3 h generally have positive values for PC1 and 6–12 h have mostly negative values with 4 h samples being clustered close to 0. Analysis of loadings (**Supplementary Figures S6**, **S7**) show changes associated with wavelengths 1200–900 cm^-1^ associated with polysaccharides and wavelengths 1600–1250 cm^-1^ associated with amides, side chain proteins and nucleic acids. PC2 accounted for 7.43 (*Lotus*) and 12.59% (clover) of the data and showed no consistent clustering or patterns for either BFT or RC. MANOVA Wilks’s lambda results also showed that the changes in plant chemistry seen as incubation time progressed were significant for BFT (*P* < 0.05). For RC, whilst changes were apparent, the MANOVA Wilk’s test results showed that these were not significant (*P* = 0.191) across the incubation times.

## Discussion

Understanding the interactions between fresh forages and the rumen microbiome post-ingestion is crucial to understanding the bottlenecks of nutrient bioavailability to the animal and ultimately develop novel technologies to increase nutrient use efficiency in grazing ruminants. In this study we investigated temporal bacterial colonization of *Lolium perenne* (PRG), *Lotus corniculatus* (BFT), and *Trifolium pratense* (RC) using both microbial and plant based ‘omic analysis. We show that colonization of PRG, BFT and RC is different, with primary to secondary colonization events being up to (primary) and post (secondary) 4 h on PRG whilst these changes occurred later on BTF and RC, with primary colonization being up to 6 h and secondary post 6 h. The changes from primary to secondary colonization were concomitant with differential changes in the chemistry of plant. The data aligns with our previous data on colonization of PRG (Huws et al., 2013, 2016; Mayorga et al., 2016) and allows comparative investigations into colonization of BFT and RC, common forages fed to, or encountered by ruminants whilst grazing.

In terms of dry matter disappearance, all plants showed a decrease in biomass over time as it was broken down and utilized by the rumen microbiome. The biggest difference identified between the forages was the overall decreased dry matter loss of BFT, compared to RC and PRG. This is most likely due to the high quantity of condensed tannins present in BFT, which have an inhibitory effect on the growth and nutrient utilization of rumen bacteria (Min et al., 2005). The rapid dry matter loss in RC in the initial 12 h, compared to PRG, is likely due to the chemical differences between them.

Irrespective of forage type or incubation time, the attached microbiota was dominated by *Anaeroplasma*, *Butyrivibrio*, *Olsenella*, *Prevotella*, *Pseudobutyrivibrio*, and *Ruminococcus*. Piao et al. (2014) found similar results when investing colonization of switchgrass by rumen bacteria, as did Belanche et al. (2017) when investigating colonization of fresh perennial ryegrass or hay. Indeed, our previous studies also corroborate that these are the dominant PRG attached bacteria irrespective of time (Huws et al., 2016; Mayorga et al., 2016). Overall, there were few significant differences between the forages in terms of the abundances of the attached bacteria when the mean was taken for each genus over time on each forage, with only *Butyrivibrio* and *Pseudobutyrivibrio* showing significance based on forage type. The attached abundances of both *Butyrivibrio* and *Pseudobutyrivibrio* were lower on BFT compared with abundances attached to PRG and RC, which may account for the reduced degradability of BFT as *Butyrivibrio* and *Pseudobutyrivibrio* spp. typically possess glycosyl hydrolases for complex carbohydrate degradation (Krause et al., 2003). There was also a tendency that *Butyrivibrio* read abundances were higher on RC, compared with PRG, a phenomenon that we have also previously found (Huws et al., 2010). Comparing within incubation time revealed clear differences in the relative abundances of the attached bacteria between the forages tested. The two major differences identified in attached bacterial abundances were those between pre-incubation (0 h) and initial colonization (1 h) and the change from primary to secondary colonization; primary colonization being up to 4 h and secondary after 4 h for PRG colonization and up to 6 h and post 6 h for BFT and RC. With respect to changes between 0 to 1 h, these were expected as the plant epiphytic bacteria are out-competed by the rumen bacteria (Huws et al., 2016). *Pseudobutyrivibrio* and *Anaeroplasma* were also more prevalent during secondary colonization timepoints on all forages. Our data for all forages broadly corroborate the data obtained by Belanche et al. (2017) when investigating colonization fresh perennial ryegrass or hay within the rumen. Belanche et al. (2017) reported that primary perennial ryegrass and hay-attached bacterial communities were dominated by *Prevotella* and *Streptococcus*, and secondary colonization was followed by an increase in abundance of *Butyrivibrio* and *Pseudobutyrivibrio*. Likewise, Piao et al. (2014) noted increases in *Pseudobutyrivibrio* and *Ruminococcus* spp. during secondary colonization of switchgrass incubated within the rumen.

Function based inferences using CowPI analysis suggested that amino acid metabolism was at it’s lowest in the BFT attached bacteria following 8 h of colonization, and significantly so in comparison with bacteria attached to RC and PRG at both 2 and 24 h. Carbohydrate metabolism was higher following 6-8 h of incubation, compared with earlier timepoints for all the forages, thus carbohydrate metabolism is more prominent during secondary colonization. Principal component analysis of FTIR spectra from all of the samples showed that the major source of variance in the data along PC axis1 were due to the fundamental differences between monocots and dicots, which was as expected. In contrast, FTIR analysis within forages gave an insight into plant chemistry changes over incubation time. It should be noted that initial changes in plant chemistry may also be influenced by plant proteases acting on the forage as the plant breaks down (Kingston-Smith et al., 2008). Irrespective of whether the variation is due to plant cell death processes, or as a consequence of bacterial metabolism, PRG showed clear clustering in terms of plant chemistry with 1, 2, 3 h samples clustering together, followed by a chemical change at 4, 6, 8 h concomitant with attached bacteria changes as primary and secondary colonization ensues. The changes in plant chemistry are attributable to polysaccharides, protein, fatty acids, amino acids, and nucleic acids. BFT and RC showed a clear pattern in terms of chemical changes with 1, 2, 3 h clustering together and 6 and 8 h clustering together, and 4 h being an intermediate. This data is in line with the previous 16S rRNA gene data findings, with respect to the primary and secondary succession, as the significant change occurs at around the same time as the proportions of colonizing bacteria changes (primary colonization up to 6 h and secondary colonization post 6 h). CowPI bacterial inference data showed no differences in lipid metabolism between forages or across time points, with the most profound differences being an increase in carbohydrate metabolism from post 6–8 h of colonization. The DM data also suggests that polysaccharide degradation increases with incubation time.

## Conclusion

This study demonstrated that the attached bacteria on PRG, BFT and RC were similar in composition and relative abundance, but temporal differences were observed at different incubations times between the forages. Changes between primary to secondary colonization events were up to (primary) and post (secondary) 4 h on PRG, whilst they occurred post 6 h on BFT and RC. Whilst CowPI predicts the function of the attached bacteria, this is by inference and by no means perfect and future studies should focus on the metatranscriptome to understand the fundamental challenges that the bacterium faces when trying to degrade the plant material. Irrespective, these data build upon our understanding on plant degradation by the rumen microbiome over time.

## Author Contributions

SH, JE, and AK-S conceived the project. KM, JE, PR-S, MS, and TW completed the laboratory work under the supervision of SH. CE completed all the analysis and wrote the paper with guidance from SH and the other co-authors. GA helped CE with FTIR and chemometric analysis.

## Conflict of Interest Statement

The authors declare that the research was conducted in the absence of any commercial or financial relationships that could be construed as a potential conflict of interest.

## Supplementary Material

FIGURE S1 Principal coordinates analysis (PCoA) of the attached bacterial genera present on *Lolium perenne*, *Lotus corniculatus*, and *Trifolium pratense* over the 1–24 h incubation period. Time in the legends refers to hours.

FIGURE S2 Constrained analysis of proximities ordination plots of the attached bacterial genera attached to all forages **(A)**, *Lolium perenne* **(B)**, *Lotus corniculatus*, **(C)** and *Trifolium pratense* **(D)** over the 1–24 h incubation period. Red circles show early colonization events and blue circles secondary colonization events. Outliers can be seen outside the circles denoted. The time legend applies to all figures.

FIGURE S3 Relative abundances of the minor colonizing bacterial genera attached to *Lolium perenne, Lotus corniculatus*, and *Trifolium pratense in vitro* rumen incubation. The *x* axis describes the type of forage and incubation time. These minor colonizers represent < 0.1% of total reads on average across time points The prefix u. signifies that the genus could not be classified with 85% confidence so family level of classification is shown.

FIGURE S4 Alpha diversity indices plotted using phyloseq Bioconductor package in R.

FIGURE S5 FTIR normalized spectra showing the change in signal intensity (absorbance) for *Lolium perenne* as a function of incubation time. Spectral data are from 60 spectra from three analytical replicates and at least two spectral analyses.

FIGURE S6 FTIR normalized spectra showing the change in signal intensity (absorbance) for *Lotus corniculatus* as a function of incubation time. Spectral data are from 60 spectra from three analytical replicates and at least two spectral analyses.

FIGURE S7 FTIR normalized spectra showing the change in signal intensity (absorbance) for *Trifolium pratense* as a function of incubation time. Spectral data are from 60 spectra from three analytical replicates and at least two spectral analyses.

TABLE S1 Sequencing information.

TABLE S2 Proportion (% of total reads) of bacterial genera attached to *Lolium perenne* (PRG), *Lotus corniculatus* (BFT) and *Trifolium pratense* (RC) at different timepoints of *in vitro* rumen incubation. Means (*n* = 3) are shown as % normalized values with P being calculated using an ANOVA. F, T, and F^∗^T signify forage type, time and forage type × time respectively. The prefix u. signifies that the genus could not be classified with 85% confidence so family level of classification is shown. NS is shown when data is non significant (*P* > 0.05).
