# Supplementary figures and images for: Using ‘Omic Approaches to Compare Temporal Bacterial Colonization of Lolium perenne, Lotus corniculatus, and Trifolium pratense in the Rumen
Source: Front Microbiol. 2018 Sep 19;9:2184. doi: 10.3389/fmicb.2018.02184 (PMC6156263; doi:10.3389/fmicb.2018.02184)

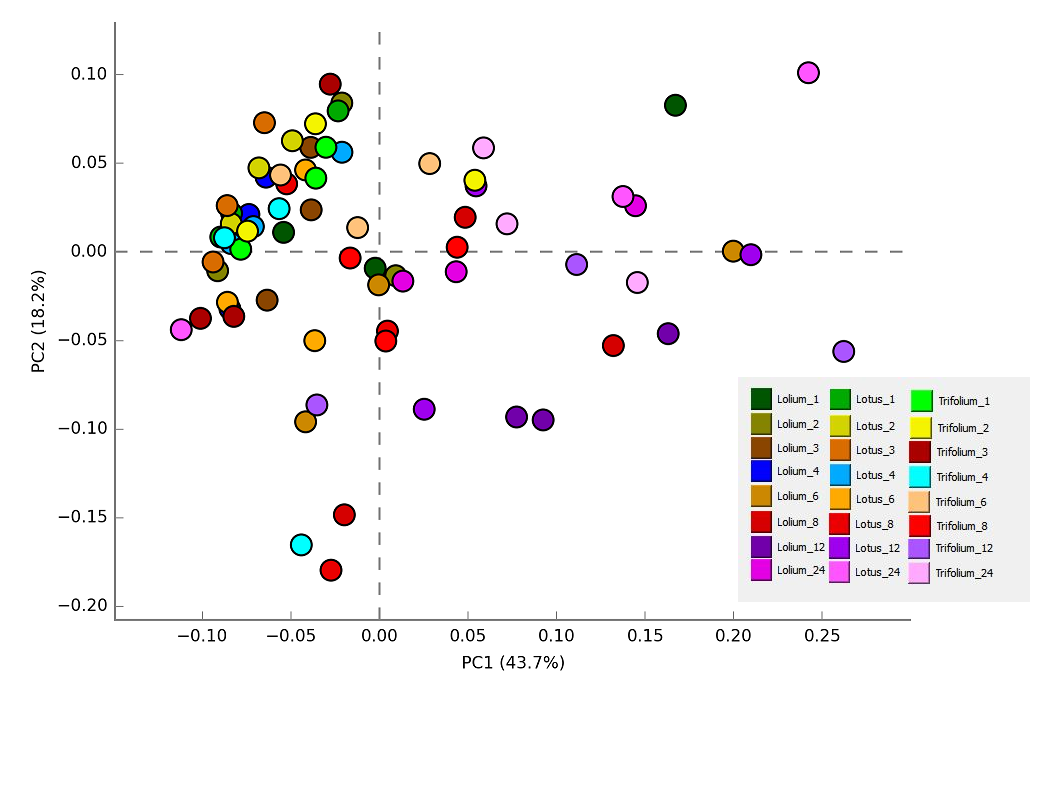

Supplement: FIGURE S1 — Principal coordinates analysis (PCoA) of the attached bacterial genera present on Lolium perenne, Lotus corniculatus, and Trifolium pratense over the 1–24 h incubation period. Time in the legends refers to hours. [file Image_1.TIF]

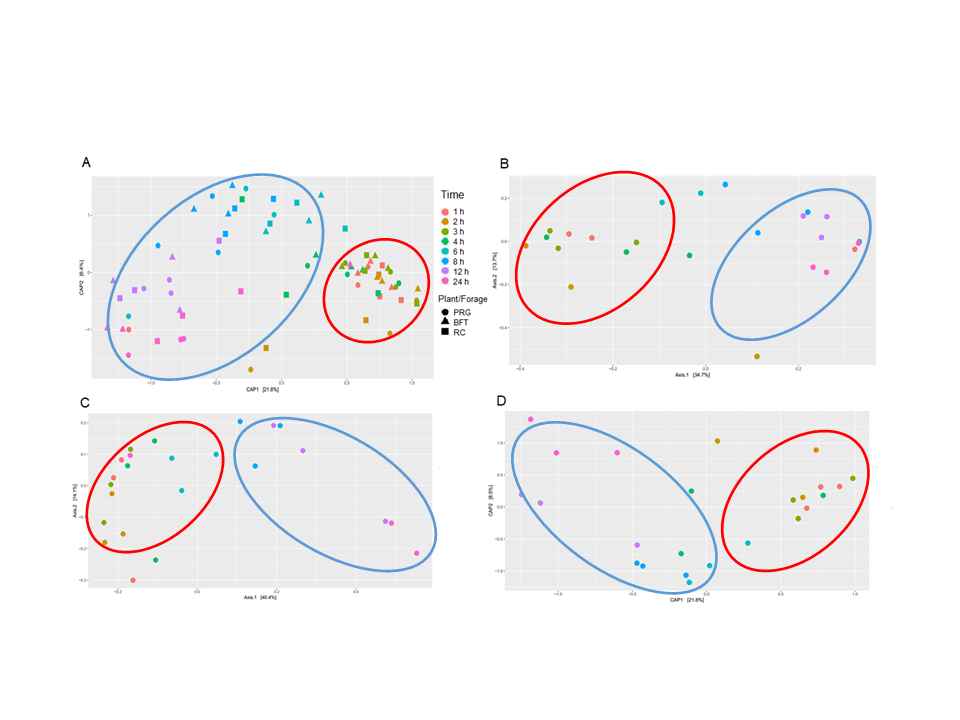

Supplement: FIGURE S2 — Constrained analysis of proximities ordination plots of the attached bacterial genera attached to all forages (A), Lolium perenne (B), Lotus corniculatus, (C) and Trifolium pratense (D) over the 1–24 h incubation period. Red circles show early colonization events and blue circles secondary colonization events. Outliers can be seen outside the circles denoted. The time legend applies to all figures. [file Image_2.TIF]

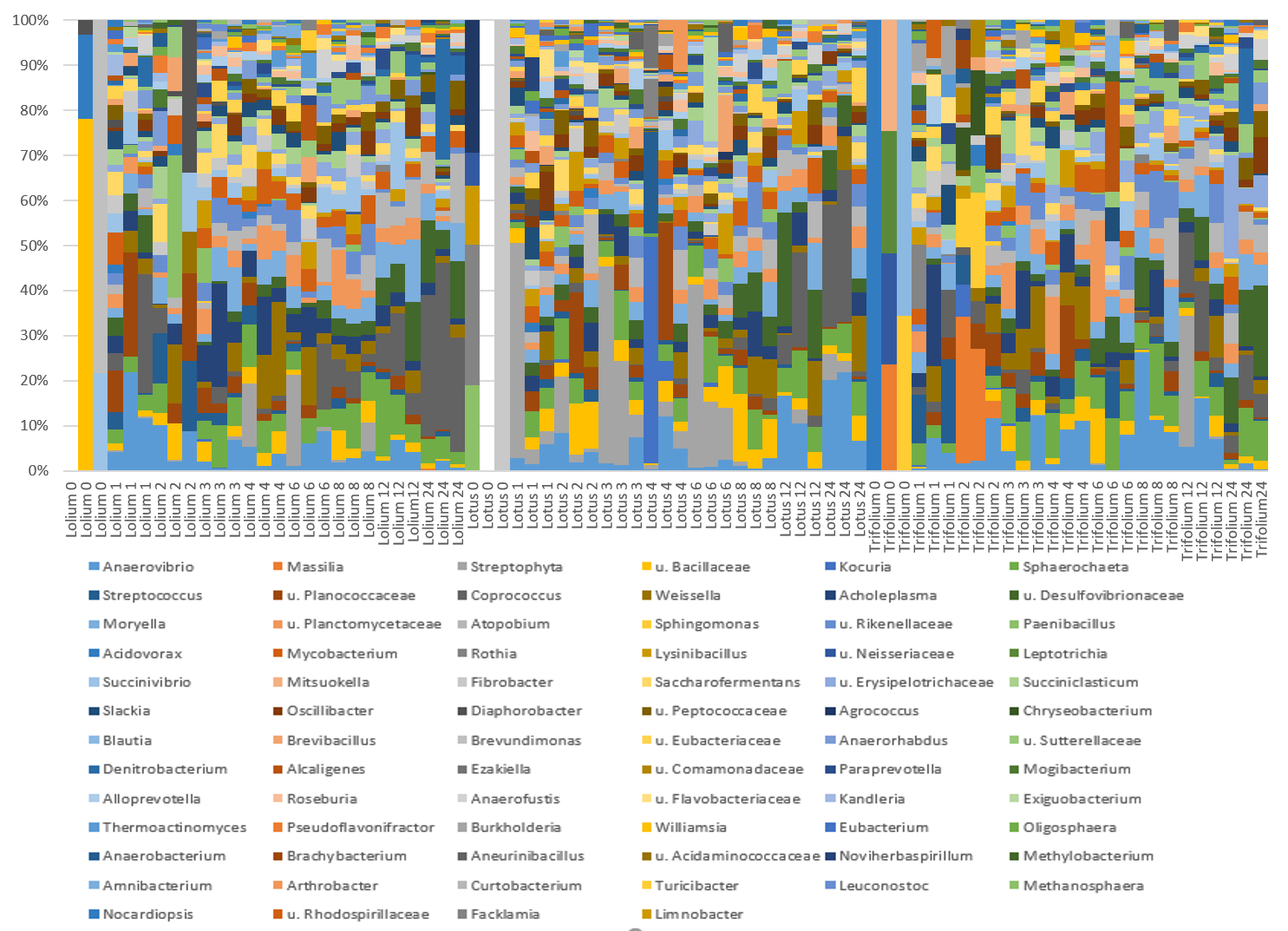

Supplement: FIGURE S3 — Relative abundances of the minor colonizing bacterial genera attached to Lolium perenne, Lotus corniculatus, and Trifolium pratense in vitro rumen incubation. The x axis describes the type of forage and incubation time. These minor colonizers represent < 0.1% of total reads on average across time points The prefix u. signifies that the genus could not be classified with 85% confidence so family level of classification is shown. [file Image_3.TIF]

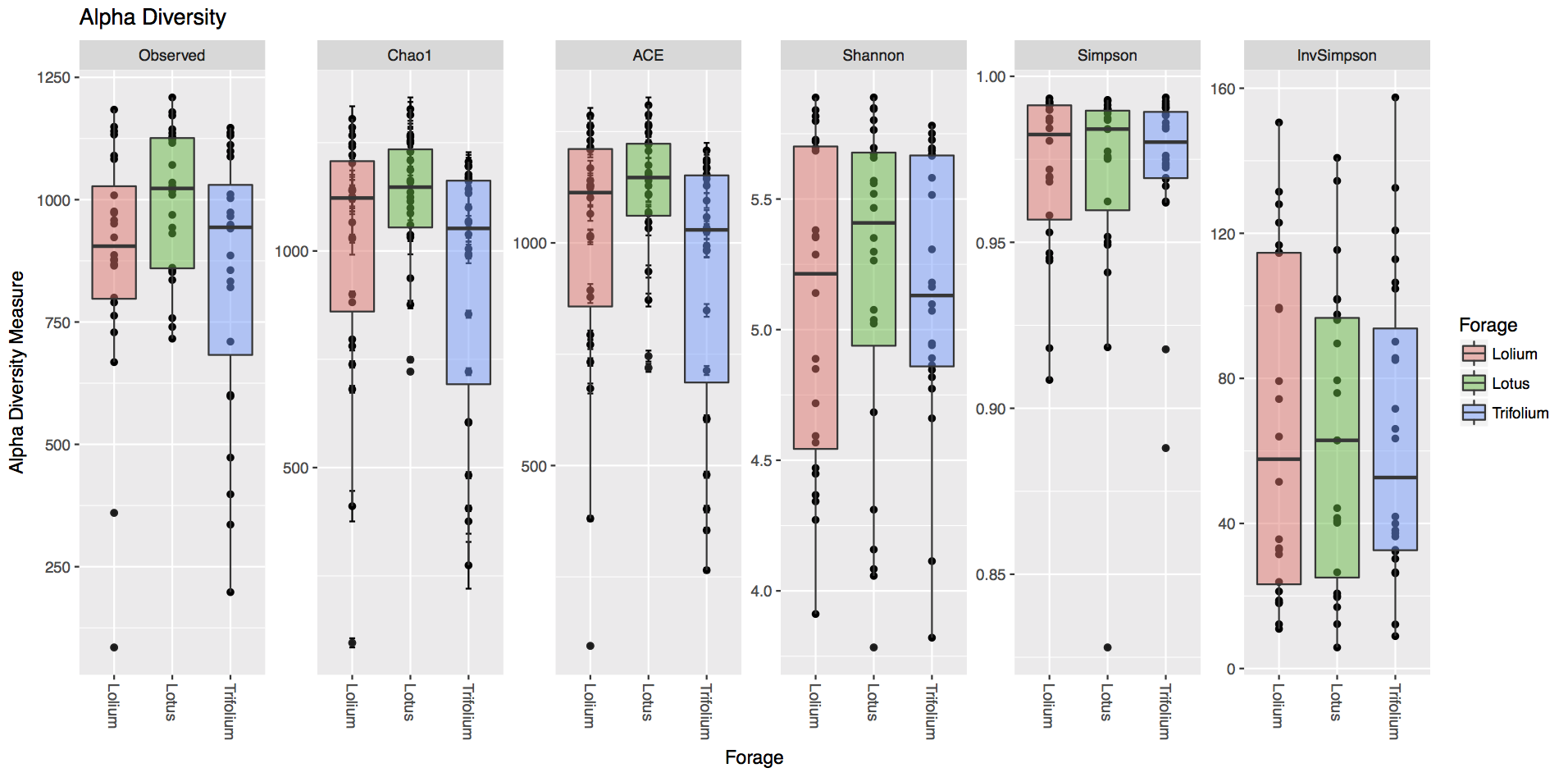

Supplement: FIGURE S4 — Alpha diversity indices plotted using phyloseq Bioconductor package in R. [file Image_4.TIF]

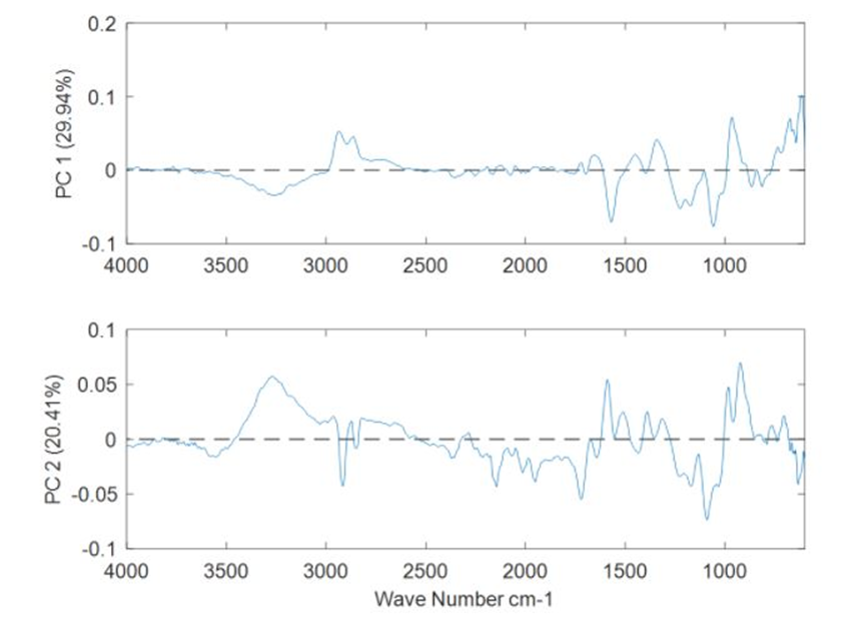

Supplement: FIGURE S5 — FTIR normalized spectra showing the change in signal intensity (absorbance) for Lolium perenne as a function of incubation time. Spectral data are from 60 spectra from three analytical replicates and at least two spectral analyses. [file Image_5.TIF]

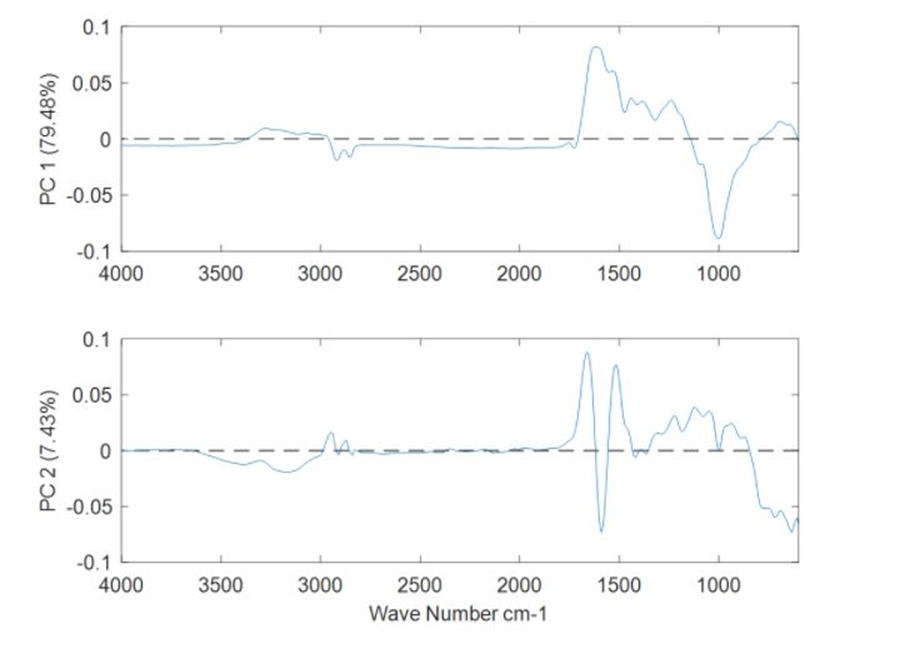

Supplement: FIGURE S6 — FTIR normalized spectra showing the change in signal intensity (absorbance) for Lotus corniculatus as a function of incubation time. Spectral data are from 60 spectra from three analytical replicates and at least two spectral analyses. [file Image_6.TIF]

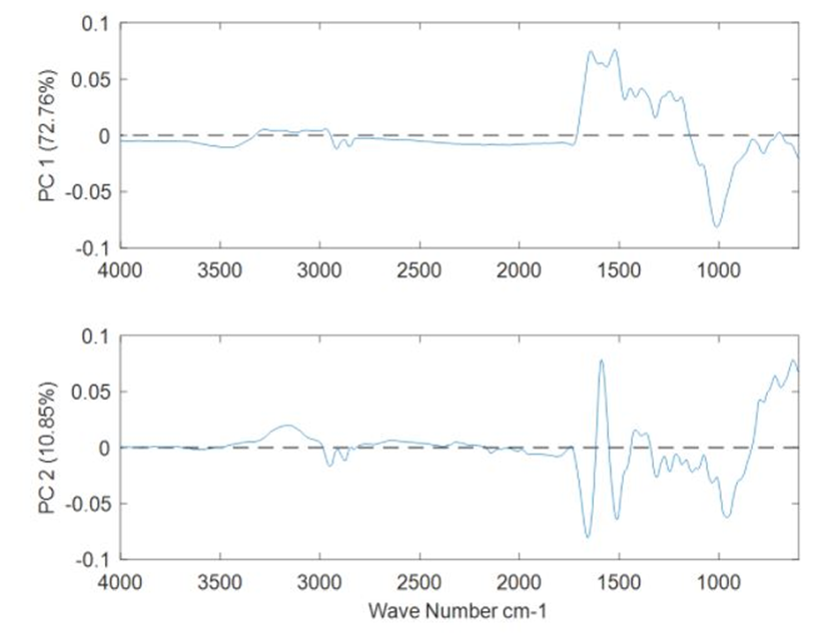

Supplement: FIGURE S7 — FTIR normalized spectra showing the change in signal intensity (absorbance) for Trifolium pratense as a function of incubation time. Spectral data are from 60 spectra from three analytical replicates and at least two spectral analyses. [file Image_7.TIF]
