# Supplementary material for: Using ‘Omic Approaches to Compare Temporal Bacterial Colonization of Lolium perenne, Lotus corniculatus, and Trifolium pratense in the Rumen
Source: Front Microbiol. 2018 Sep 19;9:2184. doi: 10.3389/fmicb.2018.02184 (PMC6156263; doi:10.3389/fmicb.2018.02184)
Supplement: TABLE S1 — Sequencing information. [file Table_1.DOCX]

**Table 1 Supplementary.** Sequencing information

| **Sequence information** | **Average (reads)** |
| --- | --- |
| Pre QC: Base pair (bp) Count | 1,882,204,533 (6,337,389) |
| Post QC: bp Count | 1,239.506,304 (4,173,422) |
| Post QC: bp count/sample | 17,215,366 (57,964) |
| Post QC: Mean Sequence Length (bp) | 304 |
