# Supplementary material for: Using ‘Omic Approaches to Compare Temporal Bacterial Colonization of Lolium perenne, Lotus corniculatus, and Trifolium pratense in the Rumen
Source: Front Microbiol. 2018 Sep 19;9:2184. doi: 10.3389/fmicb.2018.02184 (PMC6156263; doi:10.3389/fmicb.2018.02184)
Supplement: TABLE S2 — Proportion (% of total reads) of bacterial genera attached to Lolium perenne (PRG), Lotus corniculatus (BFT) and Trifolium pratense (RC) at different timepoints of in vitro rumen incubation. Means (n = 3) are shown as % normalized values with P being calculated using an ANOVA. F, T, and F∗T signify forage type, time and forage type × time respectively. The prefix u. signifies that the genus could not be classified with 85% confidence so family level of classification is shown. NS is shown when data is non significant (P > 0.05). [file Table_2.DOCX]

**Table 2 supplementary.** Proportion (% of total reads) of bacterial genera attached to *Lolium perenne* (PRG)*, Lotus corniculatus* (BFT) and *Trifolium pratense* (RC) at different timepoints of *in vitro* rumen incubation. Means (n=3) are shown as % normalised values with P being calculated using an ANOVA. F, T and F*T signify forage type, time and forage type x time respectively. The prefix u. signifies that the genus could not be classified with 85% confidence so family level of classification is shown. NS is shown when data is non significant (P>0.05).

| Genus | PRG (h) | | | | | | | | | BFT (h) | | | | | | | | | RC (h) | | | | | | | | | p value | | | SED | | |
| --- | --- | --- | --- | --- | --- | --- | --- | --- | --- | --- | --- | --- | --- | --- | --- | --- | --- | --- | --- | --- | --- | --- | --- | --- | --- | --- | --- | --- | --- | --- | --- | --- | --- |
|  | 0 | 1 | 2 | 3 | 4 | 6 | 8 | 12 | 24 | 0 | 1 | 2 | 3 | 4 | 6 | 8 | 12 | 24 | 0 | 1 | 2 | 3 | 4 | 6 | 8 | 12 | 24 | F | T | F*T | F | T | F*T |
| *Acholeplasma* | 0.00 | 0.07 | 0.05 | 0.25 | 0.14 | 0.08 | 0.02 | 0.03 | 0.03 | 0.00 | 0.07 | 0.05 | 0.10 | 0.02 | 0.03 | 0.07 | 0.02 | 0.05 | 0.00 | 0.19 | 0.01 | 0.15 | 0.11 | 0.03 | 0.07 | 0.03 | 0.02 | NS | 0.01 | NS | 0.02 | 0.04 | 0.07 |
| *Achromobacter* | 2.51 | 0.00 | 0.00 | 0.01 | 0.00 | 0.00 | 0.00 | 0.00 | 0.00 | 0.61 | 0.00 | 0.02 | 0.00 | 0.01 | 0.01 | 0.00 | 0.00 | 0.00 | 0.00 | 0.01 | 0.05 | 0.00 | 0.02 | 0.00 | 0.00 | 0.00 | 0.01 | NS | NS | NS | 0.22 | 0.38 | 0.65 |
| *Acidovorax* | 0.25 | 0.02 | 0.03 | 0.00 | 0.02 | 0.00 | 0.00 | 0.00 | 0.00 | 0.00 | 0.02 | 0.01 | 0.00 | 0.01 | 0.00 | 0.00 | 0.00 | 0.00 | 0.68 | 0.00 | 0.13 | 0.01 | 0.00 | 0.00 | 0.00 | 0.00 | 0.00 | NS | NS | NS | 0.07 | 0.12 | 0.20 |
| *Acinetobacter* | 3.41 | 0.12 | 0.00 | 0.00 | 0.01 | 0.20 | 0.01 | 0.01 | 0.00 | 2.91 | 0.05 | 0.05 | 0.02 | 0.18 | 0.05 | 0.00 | 0.00 | 0.01 | 1.43 | 0.02 | 0.23 | 0.05 | 0.03 | 0.00 | 0.01 | 0.00 | 0.00 | NS | <.001 | NS | 0.31 | 0.53 | 0.92 |
| *Agrococcus* | 0.00 | 0.00 | 0.00 | 0.00 | 0.00 | 0.00 | 0.00 | 0.00 | 0.00 | 0.62 | 0.00 | 0.00 | 0.00 | 0.01 | 0.01 | 0.00 | 0.00 | 0.00 | 0.00 | 0.00 | 0.00 | 0.00 | 0.00 | 0.00 | 0.00 | 0.00 | 0.00 | NS | NS | NS | 0.06 | 0.10 | 0.17 |
| *Alcaligenes* | 0.00 | 0.01 | 0.00 | 0.03 | 0.00 | 0.05 | 0.01 | 0.00 | 0.00 | 0.00 | 0.03 | 0.01 | 0.01 | 0.03 | 0.00 | 0.00 | 0.00 | 0.00 | 0.00 | 0.02 | 0.04 | 0.04 | 0.00 | 0.13 | 0.01 | 0.00 | 0.01 | NS | NS | NS | 0.01 | 0.02 | 0.04 |
| *Alloprevotella* | 0.00 | 0.02 | 0.01 | 0.03 | 0.02 | 0.00 | 0.00 | 0.00 | 0.00 | 0.00 | 0.02 | 0.01 | 0.04 | 0.01 | 0.02 | 0.02 | 0.00 | 0.01 | 0.00 | 0.05 | 0.00 | 0.03 | 0.00 | 0.01 | 0.01 | 0.00 | 0.00 | NS | 0.013 | NS | 0.01 | 0.01 | 0.02 |
| *Amnibacterium* | 0.00 | 0.00 | 0.00 | 0.00 | 0.01 | 0.01 | 0.01 | 0.00 | 0.00 | 0.00 | 0.01 | 0.00 | 0.00 | 0.00 | 0.00 | 0.00 | 0.00 | 0.00 | 0.00 | 0.01 | 0.00 | 0.00 | 0.00 | 0.04 | 0.00 | 0.00 | 0.01 | NS | NS | NS | 0.00 | 0.01 | 0.01 |
| *Anaerobacterium* | 0.00 | 0.00 | 0.02 | 0.01 | 0.00 | 0.00 | 0.00 | 0.00 | 0.00 | 0.00 | 0.00 | 0.00 | 0.00 | 0.00 | 0.00 | 0.00 | 0.00 | 0.00 | 0.00 | 0.00 | 0.08 | 0.00 | 0.00 | 0.01 | 0.01 | 0.00 | 0.00 | NS | NS | NS | 0.01 | 0.01 | 0.02 |
| *Anaerofustis* | 0.00 | 0.02 | 0.00 | 0.00 | 0.00 | 0.02 | 0.02 | 0.01 | 0.01 | 0.00 | 0.00 | 0.03 | 0.00 | 0.02 | 0.01 | 0.02 | 0.03 | 0.01 | 0.00 | 0.00 | 0.00 | 0.00 | 0.01 | 0.01 | 0.02 | 0.02 | 0.06 | NS | 0.012 | 0.045 | 0.00 | 0.01 | 0.01 |
| *Anaeroplasma* | 0.03 | 1.16 | 0.63 | 1.29 | 0.86 | 2.57 | 3.12 | 2.86 | 5.86 | 0.00 | 0.95 | 0.84 | 1.03 | 0.75 | 1.53 | 2.35 | 5.13 | 1.37 | 0.00 | 1.02 | 0.71 | 1.04 | 1.38 | 1.16 | 2.50 | 4.59 | 5.40 | NS | <.001 | NS | 0.39 | 0.67 | 1.16 |
| *Anaerorhabdus* | 0.00 | 0.02 | 0.06 | 0.02 | 0.04 | 0.02 | 0.02 | 0.01 | 0.01 | 0.00 | 0.05 | 0.02 | 0.03 | 0.03 | 0.04 | 0.01 | 0.02 | 0.02 | 0.00 | 0.00 | 0.00 | 0.01 | 0.00 | 0.02 | 0.02 | 0.02 | 0.05 | NS | NS | NS | 0.01 | 0.01 | 0.02 |
| *Anaerovibrio* | 0.00 | 0.27 | 0.23 | 0.05 | 0.07 | 0.07 | 0.03 | 0.04 | 0.01 | 0.00 | 0.08 | 0.08 | 0.07 | 0.14 | 0.03 | 0.01 | 0.09 | 0.20 | 0.00 | 0.08 | 0.16 | 0.10 | 0.16 | 0.04 | 0.26 | 0.10 | 0.01 | NS | 0.013 | 0.008 | 0.02 | 0.04 | 0.07 |
| *Anaerovorax* | 0.00 | 0.27 | 0.10 | 0.17 | 0.32 | 0.12 | 0.09 | 0.06 | 0.04 | 0.00 | 0.27 | 0.37 | 0.25 | 0.23 | 0.16 | 0.14 | 0.11 | 0.12 | 0.00 | 0.23 | 0.13 | 0.37 | 0.15 | 0.11 | 0.08 | 0.12 | 0.07 | NS | <.001 | NS | 0.03 | 0.05 | 0.08 |
| *Aneurinibacillus* | 0.00 | 0.00 | 0.00 | 0.00 | 0.00 | 0.00 | 0.00 | 0.00 | 0.00 | 0.00 | 0.01 | 0.01 | 0.02 | 0.00 | 0.05 | 0.00 | 0.00 | 0.00 | 0.00 | 0.00 | 0.00 | 0.00 | 0.00 | 0.01 | 0.02 | 0.00 | 0.01 | NS | NS | NS | 0.01 | 0.01 | 0.02 |
| *Arthrobacter* | 0.00 | 0.00 | 0.00 | 0.00 | 0.00 | 0.00 | 0.00 | 0.00 | 0.00 | 0.00 | 0.00 | 0.01 | 0.00 | 0.08 | 0.00 | 0.00 | 0.00 | 0.00 | 0.00 | 0.00 | 0.00 | 0.00 | 0.00 | 0.00 | 0.00 | 0.00 | 0.00 | 0.046 | NS | NS | 0.01 | 0.01 | 0.02 |
| *Atopobium* | 0.00 | 0.03 | 0.02 | 0.05 | 0.04 | 0.04 | 0.03 | 0.05 | 0.10 | 0.00 | 0.07 | 0.07 | 0.07 | 0.09 | 0.06 | 0.04 | 0.07 | 0.04 | 0.00 | 0.01 | 0.02 | 0.03 | 0.07 | 0.02 | 0.10 | 0.06 | 0.11 | NS | 0.008 | NS | 0.01 | 0.02 | 0.03 |
| *Bacillus* | 0.00 | 0.13 | 1.13 | 0.60 | 0.21 | 0.16 | 0.03 | 0.03 | 0.03 | 1.04 | 0.53 | 0.39 | 0.19 | 0.14 | 0.22 | 0.05 | 0.03 | 0.04 | 1.12 | 0.19 | 4.39 | 0.33 | 0.13 | 0.25 | 0.12 | 0.01 | 0.07 | NS | NS | NS | 0.43 | 0.74 | 1.27 |
| *Blautia* | 0.00 | 0.00 | 0.00 | 0.00 | 0.00 | 0.00 | 0.00 | 0.00 | 0.00 | 0.00 | 0.00 | 0.00 | 0.00 | 0.00 | 0.00 | 0.00 | 0.00 | 0.00 | 0.62 | 0.00 | 0.00 | 0.00 | 0.00 | 0.00 | 0.00 | 0.00 | 0.00 | NS | NS | NS | 0.06 | 0.10 | 0.17 |
| *Brachybacterium* | 0.00 | 0.00 | 0.00 | 0.00 | 0.00 | 0.00 | 0.00 | 0.00 | 0.00 | 0.00 | 0.00 | 0.00 | 0.00 | 0.00 | 0.00 | 0.00 | 0.00 | 0.00 | 0.00 | 0.00 | 0.15 | 0.00 | 0.00 | 0.00 | 0.00 | 0.00 | 0.00 | NS | NS | NS | 0.01 | 0.02 | 0.04 |
| *Brevibacillus* | 0.00 | 0.04 | 0.09 | 0.01 | 0.00 | 0.04 | 0.01 | 0.00 | 0.00 | 0.00 | 0.04 | 0.00 | 0.05 | 0.02 | 0.10 | 0.00 | 0.00 | 0.01 | 0.00 | 0.00 | 0.08 | 0.03 | 0.05 | 0.01 | 0.02 | 0.00 | 0.01 | NS | NS | NS | 0.02 | 0.03 | 0.05 |
| *Brevundimonas* | 0.52 | 0.00 | 0.00 | 0.00 | 0.00 | 0.00 | 0.00 | 0.00 | 0.00 | 0.00 | 0.00 | 0.00 | 0.00 | 0.00 | 0.00 | 0.00 | 0.00 | 0.00 | 0.00 | 0.02 | 0.03 | 0.03 | 0.00 | 0.00 | 0.00 | 0.00 | 0.00 | NS | NS | NS | 0.05 | 0.08 | 0.14 |
| *Bulleidia* | 0.00 | 0.07 | 0.06 | 0.16 | 0.10 | 0.09 | 0.10 | 0.05 | 0.05 | 0.73 | 0.16 | 0.12 | 0.13 | 0.12 | 0.23 | 0.07 | 0.11 | 0.03 | 0.00 | 0.09 | 0.12 | 0.10 | 0.16 | 0.39 | 0.21 | 0.10 | 0.13 | NS | NS | NS | 0.07 | 0.12 | 0.21 |
| *Burkholderia* | 0.00 | 0.00 | 0.00 | 0.02 | 0.00 | 0.00 | 0.00 | 0.00 | 0.00 | 0.00 | 0.01 | 0.01 | 0.00 | 0.02 | 0.00 | 0.00 | 0.00 | 0.00 | 0.00 | 0.10 | 0.02 | 0.04 | 0.00 | 0.00 | 0.00 | 0.00 | 0.00 | 0.041 | 0.041 | 0.029 | 0.01 | 0.01 | 0.02 |
| *Butyrivibrio* | 0.46 | 2.35 | 2.00 | 1.99 | 1.33 | 2.27 | 2.72 | 2.24 | 1.54 | 0.00 | 1.15 | 0.97 | 1.02 | 2.10 | 2.01 | 1.88 | 1.73 | 0.81 | 2.41 | 1.53 | 2.05 | 1.69 | 1.79 | 3.03 | 3.47 | 2.68 | 2.05 | 0.001 | 0.011 | NS | 0.26 | 0.45 | 0.78 |
| *Chryseobacterium* | 0.00 | 0.00 | 0.00 | 0.00 | 0.00 | 0.00 | 0.00 | 0.00 | 0.00 | 0.00 | 0.00 | 0.00 | 0.00 | 0.00 | 0.00 | 0.00 | 0.00 | 0.00 | 0.00 | 0.00 | 0.63 | 0.00 | 0.00 | 0.00 | 0.00 | 0.00 | 0.00 | NS | 0.005 | <.001 | 0.03 | 0.06 | 0.10 |
| *Cloacibacterium* | 0.32 | 0.01 | 0.00 | 0.00 | 0.00 | 0.01 | 0.00 | 0.00 | 0.00 | 2.70 | 0.00 | 0.00 | 0.00 | 0.00 | 0.00 | 0.00 | 0.00 | 0.00 | 0.00 | 0.00 | 0.00 | 0.00 | 0.07 | 0.00 | 0.00 | 0.00 | 0.00 | NS | NS | NS | 0.21 | 0.36 | 0.63 |
| *Coprococcus* | 0.00 | 0.15 | 0.04 | 0.03 | 0.01 | 0.04 | 0.04 | 0.07 | 0.43 | 0.00 | 0.02 | 0.02 | 0.00 | 0.01 | 0.01 | 0.02 | 0.08 | 0.24 | 0.00 | 0.08 | 0.18 | 0.06 | 0.00 | 0.01 | 0.04 | 0.12 | 0.11 | NS | <.001 | 0.021 | 0.02 | 0.04 | 0.06 |
| *Corynebacterium* | 1.19 | 0.05 | 0.05 | 0.02 | 0.11 | 0.02 | 0.01 | 0.01 | 0.02 | 4.11 | 0.16 | 0.06 | 0.06 | 0.11 | 0.05 | 0.05 | 0.01 | 0.02 | 0.37 | 0.03 | 0.07 | 0.04 | 0.10 | 0.05 | 0.01 | 0.00 | 0.04 | NS | <.001 | 0.034 | 0.22 | 0.38 | 0.66 |
| *Curtobacterium* | 0.00 | 0.01 | 0.00 | 0.01 | 0.00 | 0.00 | 0.00 | 0.00 | 0.00 | 0.00 | 0.00 | 0.00 | 0.00 | 0.00 | 0.00 | 0.00 | 0.01 | 0.00 | 0.00 | 0.02 | 0.00 | 0.01 | 0.01 | 0.02 | 0.00 | 0.00 | 0.00 | NS | NS | NS | 0.00 | 0.00 | 0.01 |
| *Delftia* | 6.40 | 0.09 | 1.95 | 0.07 | 0.15 | 0.03 | 0.01 | 0.00 | 0.00 | 25.64 | 0.18 | 0.06 | 0.01 | 0.06 | 0.03 | 0.01 | 0.02 | 0.00 | 9.47 | 0.29 | 0.37 | 0.04 | 0.49 | 0.08 | 0.04 | 0.00 | 0.00 | 0.002 | <.001 | <.001 | 0.56 | 0.97 | 1.68 |
| *Denitrobacterium* | 0.00 | 0.04 | 0.00 | 0.00 | 0.00 | 0.00 | 0.00 | 0.02 | 0.15 | 0.00 | 0.01 | 0.01 | 0.00 | 0.01 | 0.01 | 0.01 | 0.00 | 0.01 | 0.00 | 0.01 | 0.01 | 0.01 | 0.01 | 0.00 | 0.02 | 0.01 | 0.09 | NS | 0.032 | NS | 0.01 | 0.02 | 0.04 |
| *Diaphorobacter* | 0.04 | 0.01 | 0.53 | 0.01 | 0.00 | 0.00 | 0.00 | 0.00 | 0.00 | 0.00 | 0.03 | 0.01 | 0.00 | 0.01 | 0.00 | 0.00 | 0.00 | 0.00 | 0.00 | 0.00 | 0.00 | 0.01 | 0.00 | 0.00 | 0.00 | 0.00 | 0.00 | NS | NS | NS | 0.05 | 0.08 | 0.14 |
| *Eubacterium* | 0.00 | 0.01 | 0.00 | 0.02 | 0.01 | 0.00 | 0.00 | 0.00 | 0.00 | 0.00 | 0.04 | 0.03 | 0.01 | 0.00 | 0.01 | 0.00 | 0.01 | 0.00 | 0.00 | 0.01 | 0.01 | 0.01 | 0.01 | 0.00 | 0.00 | 0.00 | 0.00 | NS | 0.04 | NS | 0.00 | 0.01 | 0.01 |
| *Exiguobacterium* | 0.00 | 0.02 | 0.00 | 0.00 | 0.00 | 0.00 | 0.00 | 0.00 | 0.00 | 0.00 | 0.00 | 0.01 | 0.01 | 0.00 | 0.15 | 0.00 | 0.00 | 0.00 | 0.00 | 0.00 | 0.01 | 0.01 | 0.02 | 0.00 | 0.00 | 0.00 | 0.00 | NS | NS | NS | 0.01 | 0.02 | 0.04 |
| *Ezakiella* | 0.00 | 0.00 | 0.00 | 0.00 | 0.00 | 0.00 | 0.00 | 0.00 | 0.00 | 0.00 | 0.00 | 0.00 | 0.00 | 0.39 | 0.00 | 0.00 | 0.00 | 0.00 | 0.00 | 0.00 | 0.00 | 0.00 | 0.00 | 0.00 | 0.00 | 0.00 | 0.00 | NS | NS | NS | 0.04 | 0.06 | 0.11 |
| *Facklamia* | 0.00 | 0.00 | 0.00 | 0.00 | 0.00 | 0.01 | 0.00 | 0.00 | 0.00 | 0.00 | 0.00 | 0.00 | 0.00 | 0.00 | 0.00 | 0.00 | 0.00 | 0.00 | 0.00 | 0.00 | 0.04 | 0.00 | 0.00 | 0.00 | 0.00 | 0.00 | 0.00 | NS | NS | NS | 0.00 | 0.01 | 0.01 |
| *Fibrobacter* | 0.00 | 0.07 | 0.06 | 0.05 | 0.05 | 0.03 | 0.03 | 0.00 | 0.00 | 0.12 | 0.03 | 0.05 | 0.05 | 0.03 | 0.03 | 0.01 | 0.00 | 0.01 | 0.00 | 0.06 | 0.06 | 0.04 | 0.04 | 0.01 | 0.00 | 0.01 | 0.00 | NS | NS | NS | 0.01 | 0.03 | 0.04 |
| *Kandleria* | 0.00 | 0.04 | 0.01 | 0.01 | 0.01 | 0.02 | 0.00 | 0.00 | 0.00 | 0.00 | 0.05 | 0.01 | 0.01 | 0.00 | 0.02 | 0.00 | 0.00 | 0.00 | 0.00 | 0.00 | 0.01 | 0.01 | 0.02 | 0.01 | 0.01 | 0.00 | 0.00 | NS | 0.03 | NS | 0.00 | 0.01 | 0.01 |
| *Kocuria* | 0.00 | 0.00 | 0.00 | 0.01 | 0.00 | 0.00 | 0.00 | 0.00 | 0.00 | 0.00 | 0.00 | 0.00 | 0.00 | 2.13 | 0.01 | 0.00 | 0.00 | 0.00 | 0.00 | 0.00 | 0.17 | 0.00 | 0.00 | 0.00 | 0.00 | 0.00 | 0.00 | NS | NS | NS | 0.19 | 0.33 | 0.57 |
| *Lachnobacterium* | 0.00 | 2.03 | 0.13 | 0.32 | 0.39 | 2.73 | 2.62 | 8.05 | 6.75 | 0.68 | 0.40 | 0.40 | 0.17 | 0.28 | 0.45 | 2.52 | 5.25 | 6.18 | 3.80 | 0.44 | 0.39 | 0.25 | 0.91 | 0.88 | 1.11 | 2.59 | 1.60 | NS | <.001 | NS | 0.67 | 1.17 | 2.02 |
| *Lactobacillus* | 0.33 | 0.02 | 0.58 | 0.03 | 0.01 | 0.03 | 0.01 | 0.01 | 0.00 | 2.78 | 0.00 | 0.02 | 0.01 | 0.01 | 0.01 | 0.03 | 0.03 | 0.01 | 6.92 | 0.01 | 0.01 | 0.03 | 0.00 | 0.00 | 0.03 | 0.01 | 0.14 | NS | <.001 | 0.007 | 0.34 | 0.59 | 1.01 |
| *Lactococcus* | 0.00 | 0.01 | 0.00 | 0.00 | 0.07 | 0.00 | 0.00 | 0.00 | 0.00 | 5.02 | 0.00 | 0.01 | 0.00 | 0.00 | 0.00 | 0.00 | 0.00 | 0.00 | 0.00 | 0.03 | 0.00 | 0.00 | 0.00 | 0.00 | 0.00 | 0.00 | 0.00 | NS | NS | NS | 0.46 | 0.79 | 1.37 |
| *Leptotrichia* | 0.00 | 0.00 | 0.00 | 0.00 | 0.00 | 0.00 | 0.00 | 0.00 | 0.00 | 0.00 | 0.00 | 0.00 | 0.00 | 0.00 | 0.00 | 0.00 | 0.00 | 0.00 | 1.01 | 0.00 | 0.00 | 0.00 | 0.00 | 0.00 | 0.00 | 0.00 | 0.00 | NS | NS | NS | 0.09 | 0.16 | 0.27 |
| *Leuconostoc* | 0.00 | 0.00 | 0.00 | 0.02 | 0.00 | 0.00 | 0.00 | 0.00 | 0.00 | 0.00 | 0.03 | 0.01 | 0.00 | 0.00 | 0.00 | 0.00 | 0.00 | 0.00 | 0.00 | 0.00 | 0.00 | 0.01 | 0.00 | 0.00 | 0.00 | 0.00 | 0.00 | NS | NS | NS | 0.00 | 0.00 | 0.01 |
| *Limnobacter* | 0.00 | 0.00 | 0.00 | 0.00 | 0.00 | 0.00 | 0.00 | 0.00 | 0.00 | 0.00 | 0.00 | 0.00 | 0.00 | 0.00 | 0.00 | 0.00 | 0.00 | 0.00 | 0.00 | 0.00 | 0.00 | 0.00 | 0.06 | 0.00 | 0.00 | 0.00 | 0.00 | NS | NS | NS | 0.01 | 0.01 | 0.02 |
| *Lysinibacillus* | 0.00 | 0.01 | 0.00 | 0.05 | 0.05 | 0.04 | 0.02 | 0.01 | 0.01 | 0.27 | 0.08 | 0.09 | 0.04 | 0.04 | 0.08 | 0.02 | 0.01 | 0.02 | 0.00 | 0.05 | 0.07 | 0.04 | 0.08 | 0.01 | 0.01 | 0.01 | 0.02 | NS | NS | NS | 0.03 | 0.05 | 0.09 |
| *Massilia* | 0.00 | 0.00 | 0.00 | 0.00 | 0.00 | 0.00 | 0.00 | 0.00 | 0.00 | 0.00 | 0.00 | 0.00 | 0.00 | 0.00 | 0.00 | 0.00 | 0.00 | 0.00 | 0.88 | 0.00 | 1.49 | 0.00 | 0.00 | 0.00 | 0.00 | 0.00 | 0.00 | 0.018 | NS | 0.024 | 0.10 | 0.18 | 0.31 |
| *Methylobacterium* | 0.00 | 0.00 | 0.02 | 0.00 | 0.00 | 0.00 | 0.00 | 0.00 | 0.00 | 0.00 | 0.00 | 0.01 | 0.01 | 0.01 | 0.00 | 0.00 | 0.00 | 0.00 | 0.00 | 0.01 | 0.00 | 0.00 | 0.01 | 0.01 | 0.00 | 0.01 | 0.00 | NS | NS | NS | 0.00 | 0.00 | 0.01 |
| *Microbacterium* | 7.32 | 0.04 | 0.01 | 0.06 | 0.07 | 0.01 | 0.01 | 0.00 | 0.00 | 5.28 | 0.21 | 0.09 | 0.05 | 0.08 | 0.06 | 0.02 | 0.00 | 0.01 | 4.38 | 0.05 | 0.06 | 0.12 | 0.05 | 0.02 | 0.02 | 0.00 | 0.01 | NS | <.001 | NS | 0.47 | 0.81 | 1.40 |
| *Mitsuokella* | 0.00 | 0.00 | 0.00 | 0.00 | 0.00 | 0.00 | 0.00 | 0.00 | 0.00 | 0.00 | 0.00 | 0.00 | 0.00 | 0.00 | 0.00 | 0.00 | 0.00 | 0.00 | 0.92 | 0.00 | 0.00 | 0.00 | 0.00 | 0.00 | 0.00 | 0.00 | 0.00 | NS | NS | NS | 0.08 | 0.14 | 0.25 |
| *Mogibacterium* | 0.00 | 0.01 | 0.03 | 0.01 | 0.01 | 0.00 | 0.00 | 0.01 | 0.00 | 0.00 | 0.03 | 0.01 | 0.03 | 0.01 | 0.01 | 0.01 | 0.01 | 0.01 | 0.00 | 0.03 | 0.01 | 0.02 | 0.01 | 0.01 | 0.01 | 0.01 | 0.02 | NS | NS | NS | 0.01 | 0.01 | 0.02 |
| *Moryella* | 0.00 | 0.06 | 0.06 | 0.11 | 0.07 | 0.03 | 0.04 | 0.07 | 0.07 | 0.00 | 0.05 | 0.06 | 0.09 | 0.05 | 0.03 | 0.05 | 0.08 | 0.04 | 0.00 | 0.08 | 0.01 | 0.11 | 0.04 | 0.08 | 0.10 | 0.06 | 0.09 | NS | <.001 | NS | 0.01 | 0.02 | 0.03 |
| *Mycobacterium* | 0.00 | 0.06 | 0.07 | 0.07 | 0.09 | 0.05 | 0.04 | 0.02 | 0.03 | 0.00 | 0.08 | 0.05 | 0.08 | 0.07 | 0.03 | 0.04 | 0.05 | 0.03 | 0.00 | 0.03 | 0.01 | 0.04 | 0.05 | 0.05 | 0.03 | 0.02 | 0.05 | NS | 0.043 | NS | 0.01 | 0.02 | 0.04 |
| *Nocardiopsis* | 0.00 | 0.01 | 0.00 | 0.00 | 0.01 | 0.00 | 0.00 | 0.00 | 0.00 | 0.00 | 0.00 | 0.00 | 0.00 | 0.01 | 0.00 | 0.00 | 0.00 | 0.00 | 0.00 | 0.00 | 0.00 | 0.01 | 0.00 | 0.01 | 0.00 | 0.00 | 0.01 | NS | NS | NS | 0.00 | 0.00 | 0.01 |
| *Noviherbaspirillum* | 0.00 | 0.00 | 0.00 | 0.00 | 0.00 | 0.00 | 0.00 | 0.00 | 0.00 | 0.00 | 0.06 | 0.00 | 0.00 | 0.00 | 0.00 | 0.00 | 0.00 | 0.00 | 0.00 | 0.00 | 0.06 | 0.00 | 0.00 | 0.00 | 0.00 | 0.00 | 0.00 | NS | NS | NS | 0.01 | 0.01 | 0.02 |
| *Ochrobactrum* | 0.84 | 0.00 | 0.00 | 0.00 | 0.00 | 0.00 | 0.00 | 0.00 | 0.00 | 0.00 | 0.03 | 0.03 | 0.00 | 0.00 | 0.00 | 0.00 | 0.00 | 0.00 | 2.08 | 0.02 | 0.00 | 0.01 | 0.00 | 0.07 | 0.00 | 0.00 | 0.00 | NS | 0.001 | NS | 0.13 | 0.23 | 0.40 |
| *Oligosphaera* | 0.00 | 0.00 | 0.01 | 0.00 | 0.01 | 0.01 | 0.01 | 0.00 | 0.01 | 0.00 | 0.02 | 0.01 | 0.01 | 0.00 | 0.01 | 0.00 | 0.00 | 0.00 | 0.00 | 0.01 | 0.00 | 0.01 | 0.01 | 0.00 | 0.01 | 0.00 | 0.00 | NS | NS | NS | 0.00 | 0.01 | 0.01 |
| *Olsenella* | 0.03 | 1.64 | 1.69 | 2.98 | 4.13 | 3.43 | 2.98 | 3.75 | 3.38 | 0.00 | 3.69 | 3.97 | 4.41 | 3.76 | 3.44 | 3.26 | 2.63 | 1.86 | 0.69 | 2.74 | 1.75 | 2.52 | 1.85 | 3.55 | 2.75 | 2.81 | 1.94 | NS | <.001 | NS | 0.33 | 0.56 | 0.98 |
| *Oscillibacter* | 0.00 | 0.02 | 0.01 | 0.03 | 0.04 | 0.03 | 0.03 | 0.02 | 0.01 | 0.00 | 0.08 | 0.04 | 0.05 | 0.03 | 0.02 | 0.02 | 0.02 | 0.03 | 0.00 | 0.01 | 0.04 | 0.01 | 0.02 | 0.02 | 0.01 | 0.01 | 0.08 | NS | NS | NS | 0.01 | 0.01 | 0.02 |
| *Paenibacillus* | 0.00 | 0.02 | 0.34 | 0.06 | 0.00 | 0.00 | 0.01 | 0.00 | 0.00 | 0.40 | 0.03 | 0.04 | 0.01 | 0.01 | 0.04 | 0.01 | 0.00 | 0.01 | 0.00 | 0.00 | 0.17 | 0.02 | 0.00 | 0.01 | 0.00 | 0.00 | 0.00 | NS | NS | NS | 0.05 | 0.09 | 0.15 |
| *Paraprevotella* | 0.00 | 0.00 | 0.01 | 0.03 | 0.02 | 0.02 | 0.01 | 0.02 | 0.01 | 0.00 | 0.01 | 0.02 | 0.02 | 0.01 | 0.03 | 0.02 | 0.01 | 0.00 | 0.00 | 0.03 | 0.01 | 0.01 | 0.03 | 0.01 | 0.01 | 0.01 | 0.02 | NS | NS | NS | 0.00 | 0.01 | 0.01 |
| *Pelomonas* | 7.51 | 0.05 | 0.76 | 0.06 | 0.07 | 0.02 | 0.01 | 0.00 | 0.01 | 9.73 | 0.06 | 0.12 | 0.02 | 0.07 | 0.03 | 0.01 | 0.01 | 0.00 | 6.17 | 0.15 | 0.55 | 0.10 | 0.13 | 0.09 | 0.03 | 0.00 | 0.02 | NS | <.001 | NS | 0.26 | 0.45 | 0.78 |
| *Prevotella* | 4.82 | 14.05 | 15.13 | 16.74 | 17.34 | 15.90 | 16.99 | 14.83 | 14.10 | 0.91 | 14.61 | 14.48 | 15.61 | 15.18 | 18.02 | 22.35 | 12.77 | 14.78 | 6.45 | 14.99 | 12.63 | 18.27 | 21.06 | 12.34 | 14.90 | 14.39 | 9.40 | NS | <.001 | NS | 1.50 | 2.60 | 4.51 |
| *Propionibacterium* | 0.91 | 0.09 | 0.22 | 0.02 | 0.01 | 0.05 | 0.01 | 0.01 | 0.01 | 2.54 | 0.07 | 0.01 | 0.00 | 0.04 | 0.02 | 0.01 | 0.00 | 0.01 | 3.80 | 0.09 | 0.38 | 0.06 | 0.03 | 0.02 | 0.02 | 0.00 | 0.00 | NS | <.001 | NS | 0.20 | 0.35 | 0.61 |
| *Pseudobutyrivibrio* | 0.57 | 4.73 | 2.86 | 1.70 | 1.39 | 11.35 | 12.09 | 19.55 | 9.07 | 1.80 | 0.91 | 0.95 | 0.69 | 1.01 | 2.71 | 5.88 | 9.71 | 7.42 | 2.98 | 1.41 | 3.21 | 0.97 | 3.16 | 2.39 | 4.18 | 15.59 | 10.22 | 0.028 | <.001 | NS | 1.30 | 2.25 | 3.90 |
| *Pseudoflavonifractor* | 0.00 | 0.01 | 0.03 | 0.01 | 0.02 | 0.01 | 0.01 | 0.01 | 0.02 | 0.00 | 0.01 | 0.00 | 0.01 | 0.01 | 0.01 | 0.02 | 0.02 | 0.00 | 0.00 | 0.00 | 0.00 | 0.00 | 0.01 | 0.01 | 0.01 | 0.01 | 0.01 | NS | NS | NS | 0.00 | 0.01 | 0.01 |
| *Pseudomonas* | 1.95 | 0.00 | 0.00 | 0.00 | 0.00 | 0.00 | 0.00 | 0.00 | 0.00 | 1.01 | 0.00 | 0.00 | 0.00 | 0.00 | 0.00 | 0.00 | 0.00 | 0.00 | 0.00 | 0.01 | 0.64 | 0.00 | 0.00 | 0.00 | 0.00 | 0.00 | 0.00 | NS | 0.009 | NS | 0.16 | 0.27 | 0.47 |
| *Ralstonia* | 24.68 | 0.33 | 3.04 | 0.43 | 0.08 | 0.37 | 0.04 | 0.03 | 0.01 | 6.34 | 0.53 | 0.51 | 0.21 | 0.30 | 0.09 | 0.04 | 0.05 | 0.02 | 7.19 | 0.53 | 1.18 | 0.59 | 0.96 | 0.53 | 0.17 | 0.01 | 0.06 | 0.048 | <.001 | 0.003 | 0.99 | 1.71 | 2.97 |
| *Rhodococcus* | 6.45 | 0.01 | 0.00 | 0.05 | 0.03 | 0.00 | 0.00 | 0.00 | 0.00 | 0.00 | 0.00 | 0.01 | 0.00 | 0.01 | 0.01 | 0.00 | 0.00 | 0.00 | 0.81 | 0.00 | 0.00 | 0.00 | 0.00 | 0.06 | 0.00 | 0.00 | 0.01 | NS | NS | NS | 0.52 | 0.91 | 1.57 |
| *Roseburia* | 0.00 | 0.00 | 0.00 | 0.04 | 0.01 | 0.01 | 0.01 | 0.00 | 0.01 | 0.00 | 0.04 | 0.01 | 0.01 | 0.02 | 0.02 | 0.02 | 0.00 | 0.00 | 0.00 | 0.02 | 0.02 | 0.01 | 0.03 | 0.01 | 0.01 | 0.01 | 0.00 | NS | NS | NS | 0.00 | 0.01 | 0.01 |
| *Rothia* | 0.00 | 0.00 | 0.00 | 0.00 | 0.00 | 0.00 | 0.00 | 0.00 | 0.00 | 0.66 | 0.00 | 0.01 | 0.01 | 0.35 | 0.00 | 0.00 | 0.00 | 0.00 | 0.00 | 0.11 | 0.00 | 0.00 | 0.00 | 0.00 | 0.01 | 0.00 | 0.00 | NS | NS | NS | 0.07 | 0.12 | 0.20 |
| *Ruminococcus* | 0.03 | 2.17 | 3.04 | 2.76 | 2.99 | 1.13 | 0.89 | 1.22 | 5.00 | 0.00 | 2.28 | 1.93 | 2.14 | 1.71 | 1.66 | 1.04 | 0.57 | 1.26 | 0.82 | 2.35 | 3.02 | 3.24 | 2.42 | 1.47 | 0.78 | 0.91 | 1.94 | 0.017 | <.001 | 0.047 | 0.25 | 0.44 | 0.76 |
| *Saccharofermentans* | 0.00 | 0.07 | 0.06 | 0.10 | 0.07 | 0.03 | 0.02 | 0.02 | 0.02 | 0.00 | 0.02 | 0.05 | 0.03 | 0.01 | 0.03 | 0.02 | 0.01 | 0.02 | 0.00 | 0.07 | 0.02 | 0.10 | 0.02 | 0.03 | 0.01 | 0.01 | 0.01 | NS | 0.007 | NS | 0.01 | 0.02 | 0.03 |
| *Selenomonas* | 0.32 | 1.08 | 0.39 | 0.31 | 0.18 | 0.55 | 0.71 | 0.64 | 1.44 | 0.00 | 0.15 | 0.11 | 0.12 | 0.18 | 0.22 | 0.45 | 0.60 | 0.59 | 0.00 | 0.29 | 0.34 | 0.23 | 0.28 | 0.71 | 0.99 | 0.88 | 0.04 | <.001 | <.001 | 0.002 | 0.08 | 0.14 | 0.24 |
| *Slackia* | 0.00 | 0.05 | 0.02 | 0.03 | 0.05 | 0.02 | 0.01 | 0.01 | 0.03 | 0.00 | 0.12 | 0.01 | 0.02 | 0.04 | 0.02 | 0.02 | 0.02 | 0.01 | 0.00 | 0.07 | 0.00 | 0.02 | 0.04 | 0.06 | 0.01 | 0.02 | 0.02 | NS | <.001 | NS | 0.01 | 0.01 | 0.02 |
| *Sphaerochaeta* | 0.00 | 0.07 | 0.05 | 0.12 | 0.13 | 0.06 | 0.07 | 0.08 | 0.06 | 0.00 | 0.09 | 0.10 | 0.14 | 0.09 | 0.14 | 0.07 | 0.07 | 0.08 | 0.00 | 0.07 | 0.01 | 0.15 | 0.11 | 0.14 | 0.16 | 0.06 | 0.12 | NS | <.001 | NS | 0.02 | 0.03 | 0.05 |
| *Sphingomonas* | 0.00 | 0.00 | 0.00 | 0.00 | 0.00 | 0.00 | 0.00 | 0.00 | 0.00 | 0.00 | 0.00 | 0.01 | 0.00 | 0.00 | 0.00 | 0.00 | 0.00 | 0.00 | 0.33 | 0.00 | 0.86 | 0.00 | 0.00 | 0.00 | 0.00 | 0.00 | 0.00 | 0.031 | NS | 0.026 | 0.06 | 0.10 | 0.17 |
| *Staphylococcus* | 0.46 | 0.03 | 0.06 | 0.03 | 0.03 | 0.00 | 0.00 | 0.00 | 0.00 | 0.97 | 0.07 | 0.05 | 0.01 | 3.97 | 0.01 | 0.02 | 0.01 | 0.00 | 1.21 | 0.02 | 0.08 | 0.02 | 0.01 | 0.01 | 0.00 | 0.00 | 0.00 | NS | NS | NS | 0.39 | 0.67 | 1.16 |
| *Stenotrophomonas* | 3.00 | 0.05 | 0.66 | 0.06 | 0.04 | 0.03 | 0.01 | 0.00 | 0.00 | 9.81 | 0.06 | 0.06 | 0.01 | 0.05 | 0.02 | 0.00 | 0.00 | 0.00 | 7.49 | 0.27 | 0.78 | 0.06 | 0.12 | 0.00 | 0.00 | 0.00 | 0.01 | NS | <.001 | 0.007 | 0.35 | 0.61 | 1.05 |
| *Streptococcus* | 0.00 | 0.03 | 0.32 | 0.04 | 0.03 | 0.01 | 0.01 | 0.01 | 0.01 | 0.00 | 0.02 | 0.03 | 0.01 | 0.94 | 0.01 | 0.00 | 0.00 | 0.00 | 0.00 | 0.17 | 0.16 | 0.04 | 0.06 | 0.06 | 0.01 | 0.01 | 0.00 | NS | NS | NS | 0.09 | 0.15 | 0.26 |
| *Streptophyta* | 0.00 | 0.01 | 0.00 | 0.00 | 0.10 | 0.08 | 0.03 | 0.00 | 0.00 | 0.00 | 0.67 | 0.09 | 0.58 | 0.09 | 0.52 | 0.00 | 0.01 | 0.03 | 0.00 | 0.00 | 0.00 | 0.00 | 0.00 | 0.00 | 0.00 | 0.13 | 0.00 | 0.005 | NS | NS | 0.07 | 0.12 | 0.20 |
| *Succiniclasticum* | 0.00 | 0.03 | 0.06 | 0.04 | 0.04 | 0.02 | 0.02 | 0.01 | 0.01 | 0.00 | 0.03 | 0.01 | 0.05 | 0.02 | 0.04 | 0.02 | 0.01 | 0.02 | 0.00 | 0.07 | 0.05 | 0.07 | 0.07 | 0.02 | 0.03 | 0.01 | 0.01 | NS | 0.007 | NS | 0.01 | 0.02 | 0.03 |
| *Succinivibrio* | 0.14 | 0.09 | 0.20 | 0.05 | 0.01 | 0.02 | 0.04 | 0.05 | 0.01 | 0.00 | 0.01 | 0.01 | 0.01 | 0.04 | 0.03 | 0.02 | 0.01 | 0.01 | 0.00 | 0.03 | 0.01 | 0.04 | 0.03 | 0.04 | 0.04 | 0.03 | 0.00 | NS | NS | NS | 0.02 | 0.04 | 0.07 |
| *Thermoactinomyces* | 0.00 | 0.01 | 0.00 | 0.02 | 0.02 | 0.04 | 0.00 | 0.00 | 0.00 | 0.00 | 0.00 | 0.04 | 0.00 | 0.01 | 0.01 | 0.01 | 0.00 | 0.01 | 0.00 | 0.02 | 0.00 | 0.01 | 0.00 | 0.00 | 0.01 | 0.01 | 0.01 | NS | NS | NS | 0.01 | 0.01 | 0.02 |
| *Treponema* | 0.00 | 0.01 | 0.00 | 0.02 | 0.02 | 0.04 | 0.00 | 0.00 | 0.00 | 0.00 | 0.00 | 0.04 | 0.00 | 0.01 | 0.01 | 0.01 | 0.00 | 0.01 | 0.00 | 0.02 | 0.00 | 0.01 | 0.00 | 0.00 | 0.01 | 0.01 | 0.01 | NS | <.001 | NS | 0.01 | 0.01 | 0.02 |
| *Turicibacter* | 0.00 | 0.00 | 0.00 | 0.00 | 0.00 | 0.03 | 0.00 | 0.00 | 0.00 | 0.00 | 0.04 | 0.01 | 0.00 | 0.00 | 0.00 | 0.00 | 0.00 | 0.00 | 0.00 | 0.00 | 0.00 | 0.00 | 0.00 | 0.00 | 0.00 | 0.01 | 0.00 | NS | NS | NS | 0.00 | 0.01 | 0.01 |
| *Weissella* | 0.00 | 0.06 | 0.29 | 0.04 | 0.19 | 0.10 | 0.03 | 0.02 | 0.02 | 0.00 | 0.05 | 0.04 | 0.04 | 0.04 | 0.02 | 0.06 | 0.07 | 0.10 | 0.00 | 0.06 | 0.11 | 0.18 | 0.14 | 0.02 | 0.01 | 0.04 | 0.03 | NS | 0.012 | NS | 0.02 | 0.04 | 0.07 |
| *Williamsia* | 0.00 | 0.00 | 0.00 | 0.01 | 0.01 | 0.00 | 0.00 | 0.00 | 0.01 | 0.00 | 0.03 | 0.01 | 0.01 | 0.02 | 0.01 | 0.01 | 0.01 | 0.00 | 0.00 | 0.00 | 0.00 | 0.03 | 0.03 | 0.02 | 0.00 | 0.00 | 0.01 | NS | NS | NS | 0.00 | 0.01 | 0.01 |
| *unclassified* | 4.91 | 23.57 | 28.39 | 31.92 | 36.31 | 24.67 | 24.07 | 21.41 | 19.51 | 5.01 | 37.39 | 39.40 | 39.05 | 31.88 | 34.01 | 27.69 | 24.61 | 19.59 | 9.12 | 36.85 | 28.53 | 35.18 | 34.77 | 32.60 | 29.03 | 18.72 | 28.16 | 0.02 | <.001 | NS | 1.83 | 3.17 | 5.48 |
| *u.Acidaminococcaceae* | 0.00 | 0.01 | 0.01 | 0.00 | 0.01 | 0.01 | 0.00 | 0.00 | 0.00 | 0.00 | 0.00 | 0.01 | 0.01 | 0.00 | 0.01 | 0.00 | 0.00 | 0.00 | 0.00 | 0.02 | 0.00 | 0.01 | 0.01 | 0.00 | 0.00 | 0.00 | 0.00 | NS | NS | NS | 0.00 | 0.00 | 0.01 |
| *u.Bacillaceae* | 1.04 | 0.02 | 0.11 | 0.05 | 0.07 | 0.00 | 0.04 | 0.01 | 0.01 | 0.00 | 0.09 | 0.16 | 0.07 | 0.06 | 0.11 | 0.09 | 0.03 | 0.04 | 0.00 | 0.00 | 0.01 | 0.07 | 0.07 | 0.11 | 0.02 | 0.03 | 0.03 | NS | NS | NS | 0.10 | 0.17 | 0.29 |
| *u.Comamonadaceae* | 0.00 | 0.00 | 0.00 | 0.00 | 0.00 | 0.00 | 0.00 | 0.00 | 0.00 | 0.00 | 0.00 | 0.00 | 0.00 | 0.00 | 0.00 | 0.00 | 0.00 | 0.00 | 0.00 | 0.00 | 0.38 | 0.00 | 0.00 | 0.00 | 0.00 | 0.00 | 0.00 | 0.033 | 0.002 | <.001 | 0.02 | 0.03 | 0.05 |
| *u.Coriobacteriaceae* | 0.00 | 0.45 | 0.26 | 0.49 | 0.52 | 0.34 | 0.33 | 0.31 | 0.76 | 0.00 | 0.78 | 0.70 | 0.72 | 0.77 | 0.65 | 0.38 | 0.36 | 0.22 | 0.38 | 0.59 | 0.46 | 0.45 | 0.34 | 0.55 | 0.54 | 0.31 | 0.43 | NS | 0.011 | NS | 0.07 | 0.12 | 0.21 |
| *u.Desulfovibrionaceae* | 0.00 | 0.10 | 0.00 | 0.02 | 0.03 | 0.02 | 0.03 | 0.07 | 0.14 | 0.00 | 0.05 | 0.06 | 0.05 | 0.05 | 0.07 | 0.08 | 0.19 | 0.08 | 0.00 | 0.01 | 0.01 | 0.05 | 0.03 | 0.09 | 0.09 | 0.06 | 0.25 | NS | <.001 | 0.002 | 0.01 | 0.02 | 0.04 |
| *u.Enterobacteriaceae* | 10.83 | 0.02 | 0.26 | 0.00 | 0.02 | 0.02 | 0.01 | 0.00 | 0.01 | 0.61 | 0.04 | 0.01 | 0.01 | 0.00 | 0.01 | 0.00 | 0.00 | 0.00 | 4.50 | 0.05 | 1.37 | 0.00 | 0.02 | 0.00 | 0.00 | 0.00 | 0.02 | NS | 0.009 | NS | 0.84 | 1.46 | 2.53 |
| *u.Erysipelotrichaceae* | 0.00 | 0.02 | 0.01 | 0.03 | 0.04 | 0.02 | 0.03 | 0.01 | 0.02 | 0.00 | 0.02 | 0.02 | 0.04 | 0.03 | 0.07 | 0.02 | 0.01 | 0.02 | 0.00 | 0.01 | 0.01 | 0.02 | 0.02 | 0.08 | 0.06 | 0.03 | 0.20 | 0.043 | 0.006 | 0.011 | 0.01 | 0.02 | 0.03 |
| *u.Eubacteriaceae* | 0.00 | 0.02 | 0.00 | 0.05 | 0.02 | 0.02 | 0.01 | 0.00 | 0.00 | 0.00 | 0.05 | 0.03 | 0.02 | 0.01 | 0.03 | 0.04 | 0.01 | 0.04 | 0.00 | 0.03 | 0.03 | 0.05 | 0.03 | 0.01 | 0.02 | 0.01 | 0.00 | NS | NS | NS | 0.01 | 0.01 | 0.02 |
| *u.Flavobacteriaceae* | 0.00 | 0.00 | 0.00 | 0.01 | 0.02 | 0.02 | 0.01 | 0.01 | 0.00 | 0.00 | 0.03 | 0.02 | 0.01 | 0.00 | 0.01 | 0.00 | 0.00 | 0.01 | 0.00 | 0.06 | 0.00 | 0.02 | 0.01 | 0.01 | 0.01 | 0.01 | 0.02 | NS | NS | NS | 0.01 | 0.01 | 0.02 |
| *u.Lachnospiraceae* | 5.81 | 23.17 | 16.00 | 17.17 | 13.99 | 17.09 | 15.67 | 12.54 | 18.85 | 3.94 | 14.11 | 14.43 | 12.53 | 14.30 | 14.03 | 14.57 | 21.53 | 27.35 | 7.33 | 16.96 | 16.09 | 15.10 | 11.62 | 16.59 | 6.82 | 19.06 | 20.93 | NS | <.001 | NS | 1.38 | 2.39 | 4.14 |
| *u.Neisseriaceae* | 0.00 | 0.00 | 0.00 | 0.00 | 0.00 | 0.00 | 0.00 | 0.00 | 0.00 | 0.15 | 0.00 | 0.00 | 0.00 | 0.00 | 0.00 | 0.00 | 0.00 | 0.00 | 0.92 | 0.00 | 0.00 | 0.00 | 0.00 | 0.00 | 0.00 | 0.00 | 0.00 | NS | NS | NS | 0.08 | 0.15 | 0.25 |
| *u.Peptococcaceae* | 0.00 | 0.03 | 0.00 | 0.00 | 0.04 | 0.03 | 0.02 | 0.03 | 0.05 | 0.00 | 0.03 | 0.06 | 0.03 | 0.02 | 0.04 | 0.02 | 0.03 | 0.01 | 0.00 | 0.00 | 0.03 | 0.01 | 0.01 | 0.04 | 0.02 | 0.02 | 0.08 | NS | 0.008 | 0.036 | 0.01 | 0.01 | 0.02 |
| *u.Planctomycetaceae* | 0.00 | 0.06 | 0.02 | 0.08 | 0.08 | 0.04 | 0.06 | 0.03 | 0.02 | 0.00 | 0.04 | 0.03 | 0.05 | 0.04 | 0.07 | 0.03 | 0.03 | 0.04 | 0.00 | 0.06 | 0.01 | 0.13 | 0.10 | 0.20 | 0.05 | 0.03 | 0.06 | NS | 0.026 | NS | 0.02 | 0.03 | 0.05 |
| *u.Planococcaceae* | 0.00 | 0.27 | 0.35 | 0.07 | 0.06 | 0.02 | 0.01 | 0.02 | 0.00 | 0.00 | 0.07 | 0.19 | 0.06 | 0.22 | 0.02 | 0.03 | 0.00 | 0.01 | 0.00 | 0.04 | 0.20 | 0.08 | 0.16 | 0.01 | 0.03 | 0.01 | 0.02 | NS | 0.013 | NS | 0.04 | 0.07 | 0.12 |
| *u.Porphyromonadaceae* | 0.00 | 1.00 | 0.88 | 1.95 | 2.19 | 1.46 | 1.20 | 0.66 | 0.43 | 0.00 | 1.90 | 2.06 | 1.81 | 1.81 | 1.66 | 1.58 | 0.76 | 0.87 | 0.00 | 1.12 | 0.58 | 2.19 | 1.17 | 1.48 | 1.10 | 0.76 | 0.67 | 0.039 | <.001 | NS | 0.15 | 0.26 | 0.45 |
| *u.Prevotellaceae* | 0.12 | 1.78 | 1.37 | 1.01 | 1.38 | 1.30 | 0.95 | 1.37 | 1.64 | 0.43 | 1.52 | 1.15 | 1.34 | 1.08 | 1.39 | 1.40 | 1.15 | 3.46 | 0.12 | 0.78 | 0.55 | 1.24 | 1.43 | 1.31 | 1.07 | 1.46 | 2.61 | NS | <.001 | NS | 0.18 | 0.30 | 0.53 |
| *u.Rhodospirillaceae* | 0.00 | 0.00 | 0.00 | 0.00 | 0.00 | 0.00 | 0.00 | 0.00 | 0.00 | 0.00 | 0.00 | 0.00 | 0.00 | 0.00 | 0.00 | 0.00 | 0.00 | 0.00 | 0.00 | 0.06 | 0.00 | 0.00 | 0.00 | 0.00 | 0.00 | 0.00 | 0.00 | NS | NS | NS | 0.01 | 0.01 | 0.02 |
| *u.Ruminococcaceae* | 0.00 | 8.07 | 9.61 | 12.32 | 11.56 | 7.10 | 6.57 | 4.64 | 5.87 | 0.84 | 12.41 | 12.87 | 14.52 | 10.72 | 11.09 | 7.71 | 5.65 | 4.52 | 0.40 | 11.35 | 9.37 | 10.74 | 9.45 | 10.25 | 7.93 | 4.12 | 6.38 | NS | <.001 | NS | 0.78 | 1.36 | 2.35 |
| *u.Sutterellaceae* | 0.00 | 0.00 | 0.07 | 0.00 | 0.01 | 0.03 | 0.03 | 0.02 | 0.01 | 0.00 | 0.00 | 0.00 | 0.00 | 0.00 | 0.02 | 0.01 | 0.04 | 0.00 | 0.00 | 0.03 | 0.00 | 0.01 | 0.00 | 0.04 | 0.06 | 0.03 | 0.07 | NS | NS | NS | 0.01 | 0.01 | 0.02 |
| *u.Veillonellaceae* | 2.82 | 6.81 | 3.76 | 2.67 | 1.89 | 5.14 | 7.15 | 4.06 | 1.99 | 2.34 | 2.10 | 1.55 | 1.00 | 2.85 | 3.16 | 5.20 | 5.63 | 3.61 | 2.53 | 2.80 | 3.12 | 2.19 | 3.09 | 7.95 | 10.24 | 8.94 | 0.57 | 0.049 | <.001 | NS | 0.62 | 1.08 | 1.87 |
